# Acclimatory responses of the *Daphnia pulex *proteome to environmental changes. II. Chronic exposure to different temperatures (10 and 20°C) mainly affects protein metabolism

**DOI:** 10.1186/1472-6793-9-8

**Published:** 2009-04-21

**Authors:** Susanne Schwerin, Bettina Zeis, Tobias Lamkemeyer, Rüdiger J Paul, Marita Koch, Johannes Madlung, Claudia Fladerer, Ralph Pirow

**Affiliations:** 1 Institute of Zoophysiology, University of Münster, Münster, Germany; 2 Proteom Centrum Tübingen, Interfaculty Institute for Cell Biology, University of Tübingen, Tübingen, Germany

## Abstract

**Background:**

Temperature affects essentially every aspect of the biology of poikilothermic animals including the energy and mass budgets, activity, growth, and reproduction. While thermal effects in ecologically important groups such as daphnids have been intensively studied at the ecosystem level and at least partly at the organismic level, much less is known about the molecular mechanisms underlying the acclimation to different temperatures. By using 2D gel electrophoresis and mass spectrometry, the present study identified the major elements of the temperature-induced subset of the proteome from differently acclimated *Daphnia pulex*.

**Results:**

Specific sets of proteins were found to be differentially expressed in 10°C or 20°C acclimated *D. pulex*. Most cold-repressed proteins comprised secretory enzymes which are involved in protein digestion (trypsins, chymotrypsins, astacin, carboxypeptidases). The cold-induced sets of proteins included several vitellogenin and actin isoforms (cytoplasmic and muscle-specific), and an AAA+ ATPase. Carbohydrate-modifying enzymes were constitutively expressed or down-regulated in the cold.

**Conclusion:**

Specific sets of cold-repressed and cold-induced proteins in *D. pulex *can be related to changes in the cellular demand for amino acids or to the compensatory control of physiological processes. The increase of proteolytic enzyme concentration and the decrease of vitellogenin, actin and total protein concentration between 10°C and 20°C acclimated animals reflect the increased amino-acids demand and the reduced protein reserves in the animal's body. Conversely, the increase of actin concentration in cold-acclimated animals may contribute to a compensatory mechanism which ensures the relative constancy of muscular performance. The sheer number of peptidase genes (serine-peptidase-like: > 200, astacin-like: 36, carboxypeptidase-like: 30) in the *D. pulex *genome suggests large-scaled gene family expansions that might reflect specific adaptations to the lifestyle of a planktonic filter feeder in a highly variable aquatic environment.

## Background

Planktonic crustaceans of the genus *Daphnia *experience pronounced variations in ambient parameters such as oxygen concentration and temperature in the field and show plastic adaptive responses to these environmental changes. Differential regulation of gene expression provides specific sets of proteins for the maintenance of cellular function under altered ambient conditions. The recent release of the *Daphnia pulex *genome sequence [1,2] offers the opportunity to relate proteomic adjustments to the differentially regulated genes.

Temperature affects the performance of poikilothermic animals at all levels of biological organization ranging from biochemical reactions *via *physiological processes to organismic properties such as fecundity and reproductive success. Acute changes in water temperature, for example, have a strong effect on systemic parameters such as heart and ventilation rate of *Daphnia *spp. (e.g. [3]). However, such physiological perturbations can be damped by acclimatory processes. Previous studies [3-6] have shown that the metabolic rates, heart and ventilation rates, and muscular performances of several *Daphnia *species at 10°C and 20°C are not as different as expected from the Q_10 _rule, provided the animals have the chance to acclimate to the temperature at which they were tested. Such a type of compensatory control (metabolic cold adaptation) is primarily based on adjustments in enzyme concentration [7]. Nevertheless, a more or less reduced metabolic rate in the cold decreases the nutritive requirements [8] and causes also a retardation in somatic growth and development [9-11]. To mechanistically explain the role of temperature acclimation for the control of physiological processes, it is essential to know the adjustments which occur at the proteomic level.

The present study analyzed the protein expression patterns of 10°C and 20°C acclimated animals of *Daphnia pulex *under normoxic conditions. Two-dimensional gel electrophoresis and mass spectrometry were employed to identify the major elements of the temperature-induced subset of the proteome. Based on their putative functions, the probable physiological role of these sets of proteins are discussed.

## Results

**Figure 1:**
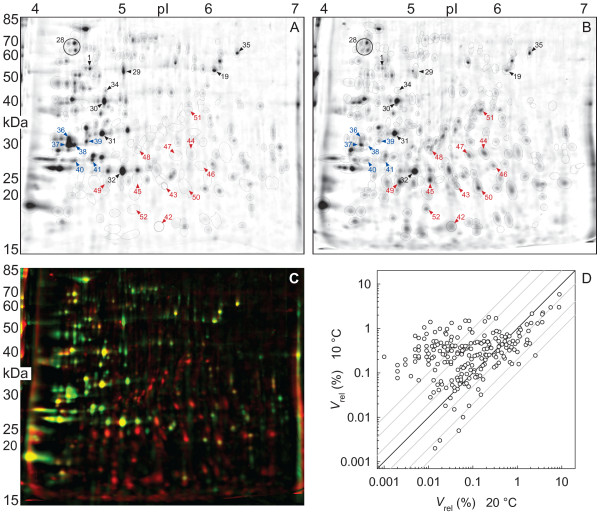
**2D protein gels from *Daphnia pulex *acclimated at 20°C (A) and 10°C (B)**. Gel images represent fusion (average) images from a set of three (A) or two (B) biological replicates. Consensus spots used for comparison in (D) are encircled. Blue and red numbers indicate cold-repressed and cold-induced protein spots that were picked from the 2D gels for mass-spectrometric analysis. Black numbers indicate previously identified proteins [12]. (C) Dual-channel representation of the gel images shown in (A) and (B). Protein spots of similar expression intensity appear in yellow. Green indicates that spots are much stronger or unique on the gel from 20°C-acclimated animals, whereas red means that spots are much stronger or unique in the gel from 10°C-acclimated *D. pulex*. (D) Scatter plot showing the comparison of expression levels in the two fusion images (*V*_rel_: relative spot volume).

Two-dimensional gels were prepared from total soluble proteins extracted from 10°C or 20°C acclimated cultures of *Daphnia pulex *kept under normoxia (oxygen partial pressure: 20 kPa). A total of 224 spots were detected in representative fusion images for each acclimation condition (Figure 1A, B; encircled spots). The dual-channel representation of both fusion gels revealed a large set of cold-induced proteins of low molecular weight (*M*_r _< 40 kDa) in the lower right diagonal half of the gel (Figure 1C; red-colored spots). Proteins of reduced expression in the cold were mainly confined to the low-pI range (pI = 4–5) in the upper left diagonal half of the gel (green-colored spots).

A total number of 17 spots comprising cold-repressed proteins (36–41, Figure 1A) and cold-induced proteins (spots 42–52, Figure 1B) were successfully identified by mass spectrometry (Tables 1, see Table 2 for corresponding protein IDs and gen models). Additionally included into the inter-gel comparison was a set of spots (1, 19–22, 28–32, 34–35), the identity of which was already known from a previous study [12]. These spots showed either constitutive or temperature-dependent expressions.

**Table 1:** Identified proteins from *Daphnia pulex *acclimated to 10°C or 20°C

**Spot no**.	**Specificity N10: N20**	**Matched peptide sequences**	**Sequence coverage^a)^**	**Mascot score^b)^**	***M*_r_gel/*M*_r_predicted**	**pI gel/pI predicted**	**SP Length**	**PP Length**	**Putative function (symbolic name)**
***Proteolytic enzymes***									

36	0.3*	1. VVAGEHSLR 2. SVDVPVVDDDTCNR	8.9%	149	30/26–29	4.4/4.4–4.8	15	27	Trypsin (**TRY5L**)
									
		1. LTAAEEPTRVEIR 2. IRNDVALIK	7.5%	80	30/25–30	4.4/4.5–5.3	18	48	Chymotrypsin (**CHY1A**)

37	0.2*	1. GVTDLTIFR 2. VVAGEHSLR 3. VVAGEHSLRTDSGLEQNR	9.8%	159	29/26–29	4.4/4.4–4.8	15	27	Trypsin (**TRY5F**)
									
		1. VVAGEHSLR 2. SVDVPVVDDDTCNR	8.9%	149	29/26–29	4.4/4.4–4.8	15	27	Trypsin (**TRY5L**)

38	0.5*	1. GLADADIAVFK 2. LIWMGQYNR 3. YYRDELAGK	10.7%	123	29/30	4.5/4.5	19		Endoribonuclease-like protein (**ERNA**)
									
		1. GLADADIAVFK 2. LIWMGQYNR 3. YYRDELAGK	8.0%	123	29/39	4.5/4.6	20		Endoribonuclease-like protein (**ERNB**)
									
		1. VVAGEHSLR 2. SVDVPVVDDDTCNR	8.9%	149	29/26–29	4.5/4.4–4.8	15	27	Trypsin (**TRY5L**)
									
		1. GVTDLTIFR 2. VVAGEHSLR	6.5%	80	29/26–29	4.5/4.4–4.8	15	27	Trypsin (**TRY5F**)

39	0.4*	1. VVAGEHSLR 2. SVDVPVVDDDTCNR	8.9%	149	29/26–29	4.6/4.4–4.8	15	27	Trypsin (**TRY5L**)
									
		1. GVTDLTIFR 2. VVAGEHSLR	6.5%	120	29/26–29	4.6/4.4–4.8	15	27	Trypsin (**TRY5F**)

40	0.6*	1. TTEEYYVSVQK 2. TGGGCYSYIGR	6.5%	112	25/23–27	4.5/4.7–4.6	?	39 ?	Astacin (**ACN2**)
									
		1. GVTDLTIFR 2. VVAGEHSLR	6.5%	109	25/26–29	4.5/4.4–4.8	15	27	Trypsin (**TRY5F**)

41	0.4*	1. LTAAEEPTR 2. LTAAEEPTRVEVR 3. IINDVALIK	9.1%	141	25/25–30	4.7/4.4–5.0	18	47	Chymotrypsin (**CHY1C**)

28	1.2	see [12]							Peptidase M13 Peptidase M2 Carboxylesterase, type B Sphingomyelin phosphodiesterase Sphingomyelin phosphodiesterase

31	0.6	see [12]			30/34–45 30/35–46 29/26–29	4.8/4.9–4.8 4.8/5.1–4.9 4.4/4.4–4.8	16 16 15	92 93 27	Carboxypeptidase A (**CPA1A**) Carboxypeptidase A (**CPA1B**) Trypsin (**TRY5F**)

32	0.3	see [12]			23/24–27	5.0/5.2–5.4	17	24	Trypsin (**TRY4B**)

***Egg yolk proteins & precursors***									

43	7.3*	see Figure 2			/190–220	/6.4–6.7	17–20		Vitellogenin (**VTG1**, **VTG2**, **VTG4**)

44	7.1*	see Figure 3 see Figure 2	15.7% 2.8%	271	25/42 25/220	5.8/5.3 5.8/6.7	17 17		Actin Vitellogenin (**VTG1**)

45	5.9*	see Figure 2	2.2%	132	21/190	5.2/6.4	20		Vitellogenin (**VTG4**)

46	5.2*	see Figure 2	2.9%	361	21/220	5.9/6.7	17		Vitellogenin (**VTG1**)

47	4.9*	see Figure 3 see Figure 2	9.6% 2.0%		25/42 25/220	5.6/5.3 5.6/6.7	17		Actin Vitellogenin (**VTG1**, **VTG2**)
									
		1. EDQMDYLEEK 2. LLVEKER 3. YSVDEELNK	3.6%		25/83	5.6/4.7			HSP90

49	4.4*	see Figure 2	?.?%	???	21/190–220	4.8/6.4–6.7	17–20		Vitellogenin (**VTG1**, **VTG2**, **VTG4**)

50	4.2*	see Figure 2	2.2%	163	20/220	5.7/6.7	17		Vitellogenin (**VTG1**)

52	3.7	see Figure 2	3.0%	344	18/220	5.1/6.7	17		Vitellogenin (**VTG1**, **VTG2**)

***Cytoskeleton & muscle proteins***									

48	4.5*	see Figure 2 see Figure 3	?.?%	???	27/42	5.2/5.3			Actin Vitellogenin (**VTG4**)
									
		1. EQLDEESEAK 2. AEELEDAKR 3. ATVLANQMEK			27/220	5.2/5.9			Myosin heavy chain (**MHC-1**)
									
		1. LTTDPAFLEK 2. NAAAVHEIR 3. GDLGIEIPPEK			27/??	5.2/?			Pyruvate kinase

51	3.6*	see Figure 3	?.?%	???	36/42	5.7/5.3			Actin

***ATPase***									

42	9.8*	2. GNEDLSTAILK 3. MDELQLFK 4. GDIFIVR 5. KQLALIK 6. EMVELPLR	5.1%	214	16/89	5.3/5.0			AAA+ ATPase

***Carbohydrate-modifying enzymes***									

35	1.2	see [12]							α-Amylase (**AMY**)

34	1.0	see [12]							Exo-β-1,3-Glucanase (**EXG5**)

1	0.4	see [12]							Cellubiohydrolase (**CEL7A**)

29	0.3	see [12]							Endo-β-1,4-Glucanase (**CEL9A**) Paramyosin (**PMY**)

30	0.6	see [12]							Endo-β-1,4-Mannanase (**MAN5A**) β-1,3-Glucan-binding protein

19	0.6	see [12]							Enolase (**ENO**)

Identification was based on 2D gel electrophoresis and nano-HPLC-ESI-MS/MS analysis of trypsin-digested proteins matched against the "Frozen Gene Catalog" of the *D. pulex *protein database [2]. The compiled information includes the spot number (Figure 1A, B), the 10-to-20°C expression ratio, the number and sequences of matched peptides, the sequence coverage, the Mascot score as a statistical measure of identification probability, the experimental and theoretical molecular weight (*M*_r_) and isolectric point (pI) of the mature protein (without signal peptide), the predicted length of the N-terminal signal peptide (SP) in secretory proteins, as well as the putative function and symbolic name of the protein. The length of the putative pro-peptide (PP) is additionally provided for proteolytic enzymes that are secrected as inactive precursors (zymogens). The predicted *M*_r _and pI values of zymogens and the mature enzymes are given as value ranges. The amino acid sequences of the identified proteins were derived from the gene models listed in Table 2. ^a)^percentage of predicted protein sequence covered by matched peptides. ^b)^Probability-based MOWSE score: -10*Log(*P*), where *P *is the probability that the observed match is a random event. Scores >38 indicate identity or extensive homology (p < 0.05). Protein scores are derived from ions scores as a non-probabilistic basis for ranking protein hits. The Mascot-score calculation was performed using whole-protein sequence (including the N-terminal signal peptide in case of extracellular proteins). *p < 0.05 (*t*-Test).

**Table 2:** List of referred proteins and gene models

**Putative function**	**Symbol**	**Model name**	**Protein ID**	**Reference ID**
Trypsin	TRY1	PIR_PASA_GEN_1500076	**347826**	301879

Trypsin	TRY2	PIR_PASA_GEN_5300037	**347779**	306771

Trypsin	TRY3	PIR_PASA_GEN_6100026	**347764**	307264

Trypsin	TRY4A	PIR_fgenesh1_pg.C_scaffold_23000179	**347804**	102943

Trypsin	TRY4B	PIR_estExt_fgenesh1_kg.C_230008	**347242**	230885

Trypsin	TRY5A	PIR_PASA_GEN_4200081	**347777**	305924

Trypsin	TRY5B	PIR_NCBI_GNO_4200123	**347775**	321745

Trypsin	TRY5C	PIR_NCBI_GNO_4200124	**347774**	106429

Trypsin	TRY5D	PIR_estExt_fgenesh1_kg.C_420021	**347772**	231151

Trypsin	TRY5E	PIR_NCBI_GNO_4200126	**347773**	321748

Trypsin	TRY5F	PIR_SNAP_00016212	**347771**	231152

Trypsin	TRY5G	PIR_NCBI_GNO_4200130	**347770**	248155

Trypsin	TRY5H	PIR_PASA_GEN_4200082	**347769**	305925

Trypsin	TRY5I	PIR_PASA_GEN_4200034	**347768**	305886

Trypsin	TRY5J	PIR_PASA_GEN_4200035	**347765**	305887

Trypsin	TRY5K	PIR_estExt_fgenesh1_kg.C_850001	**347782**	231482

Trypsin	TRY5L	PIR_NCBI_GNO_8500013	**347781**	59836

Trypsin	TRY5M	PIR_NCBI_GNO_24500018	**347780**	65745

Chymotrypsin	CHY1A	PIR_PASA_GEN_2900126	**347760**	304512

Chymotrypsin	CHY1B	PIR_NCBI_GNO_2900206	**347750**	319507

Chymotrypsin	CHY1C	PIR_NCBI_GNO_2900207	**347751**	52244

Chymotrypsin	CHY1D	PIR_PASA_GEN_2900062	**347749**	52244

Chymotrypsin	CHY1E	PIR_PASA_GEN_2900063	**347752**	304463

Chymotrypsin	CHY1F	PIR_NCBI_GNO_2900210	**347753**	26258

Chymotrypsin	CHY1G	PIR_PASA_GEN_2900130	**347754**	304515

Chymotrypsin	CHY1H	PIR_estExt_fgenesh1_kg.C_290019	**347757**	231027

Endoribonuclease	ERNA	PIR_PASA_GEN_12200001	**347694**	301221

Endoribonuclease	ERNB	PIR_PASA_GEN_6000032	**347697**	307196

Astacin	ACN2	PIR_NCBI_GNO_18200007	**347841**	93694

Peptidase M13		estExt_Genewise1Plus.C_750105	200882	200882

Peptidase M2		PASA_GEN_6000071	307230	307230

Carboxylesterase, type B		PASA_GEN_25200006	304160	304160

Sphingomyelin phosphodiesterase		PASA_GEN_2900053	304453	304453

Sphingomyelin phosphodiesterase		PASA_GEN_13800028	301526	301526

Carboxypeptidase A	CPA1A	estExt_Genewise1Plus.C_150058	195011	195011

Carboxypeptidase A	CPA1B	NCBI_GNO_1500041	315693	315693

Vitellogenin	VTG1	BDE_estExt_Genewise1.C_9580001	**299677**	219769

Vitellogenin	VTG2	PIR_estExt_fgenesh1_pg.C_9580001	**347667**	229959

Vitellogenin	VTG2	PIR_estExt_fgenesh1_pg.C_470029	**347678**	226075

Vitellogenin	VTG3	estExt_fgenesh1_pg.C_470021	226068	226068

Vitellogenin	VTG4	PASA_GEN_8300068	308693	308693

Actin	ACT1A	PIR_PASA_GEN_0400163	**347740**	305550

Actin	ACT1B	estExt_fgenesh1_pg.C_1190006	**228751**	301040

Actin	ACT1C	PIR_PASA_GEN_0500107	**347742**	306442

Actin	ACT1D	PIR_fgenesh1_pm.C_scaffold_66000006	**347743**	129328

Actin	ACT2A	PIR_PASA_GEN_0100278	**347739**	300012

Actin	ACT2B	PIR_estExt_Genewise1Plus.C_20413	**347736**	190689

Actin	ACT2C	PIR_e_gw1.2.692.1	**347703**	40361

Myosin	MHC-1	PIR_7_PIR_NCBI_GNO_0600448	**347733**	192727

Myosin	PMY	PIR_estExt_Genewise1.C_2380001	**347700**	219409

HSP90		PASA_GEN_17300027	302452	302452

Pyruvate kinase		NCBI_GNO_29900007	334106	334106

AAA+ ATPase		PASA_GEN_8000045	308570	308570

α-Amylase	AMY	FRA_PASA_GEN_2100059	**347603**	303445

Exo-β-1,3-glucanase	EXG5	PIR_PASA_GEN_1000289	**347606**	300436

Cellubiohydrolase	CEL7A	PIR_PASA_GEN_1000209	**347598**	300366

Endo-β-1,4-glucanase	CEL9A	PIR_estExt_fgenesh1_kg.C_70001	**347602**	230437

β-1,3-glucan-binding protein		PASA_GEN_0200102	303036	303036

Endo-β-1,4-mannanase	MAN5A	PIR_PASA_GEN_8600009	**347627**	308762

Enolase	ENO	PIR_PASA_GEN_1500033	**347595**	301844

Putative functions and symbolic names of identified proteins are given in relation to gene model names and protein identification numbers of those loci which were referred to in the present study. DappuDraft protein IDs in bold type indicate manually curated gene models that may differ from those contained in the 'Filtered Models v1.1' set (released by the Joint Genome Institute in July 2007). The Reference DappuDraft gene ID can be used to retrieve the corresponding models from the Filtered Models set.

It is conspicuous, that a separation of cold-induced and cold-repressed proteins by *M*_r_/pI leads to protein groups of similar classification. Almost all of the identified proteins with a reduced expression in the cold (expression reduction by 40–80%) were secretory enzymes involved in protein digestion (spots 31–32 and 36–41, Table 1). These include three trypsins (TRY4B, TRY5F, TRY5L), two chymotrypsins (CHY1A, CHY1C), one astacin (ACN2), and two carboxypeptidases (CPA1A, CPA1B). All these proteins are synthesized as pro-enzymes (zymogens), which are activated by the removal of an N-terminal propeptide (3–11 kDa). Owing to the similarities in their *M*_r_/pI values, these proteins were multiply identified among the analysed spots. In addition, the multiple occurrence of TRY5F and CHY1C in spots with assigned *M*_r _values of 25 and ≈ 30 kDa may be explained by the possible co-presence of pro-enzymes and enzymes. The only non-proteolytic proteins identified among these spots were two secretory proteins (ERNA, ERNB) carrying the characteristic domain of the EndoU/XendoU family of endoribonucleases [13,14]. The spot region 28, which was excised and analyzed in a previous study [12], contained a mixture of enzymes (including peptidases of the family M2 and M13), which made an expression evaluation impossible.

**Figure 2:**
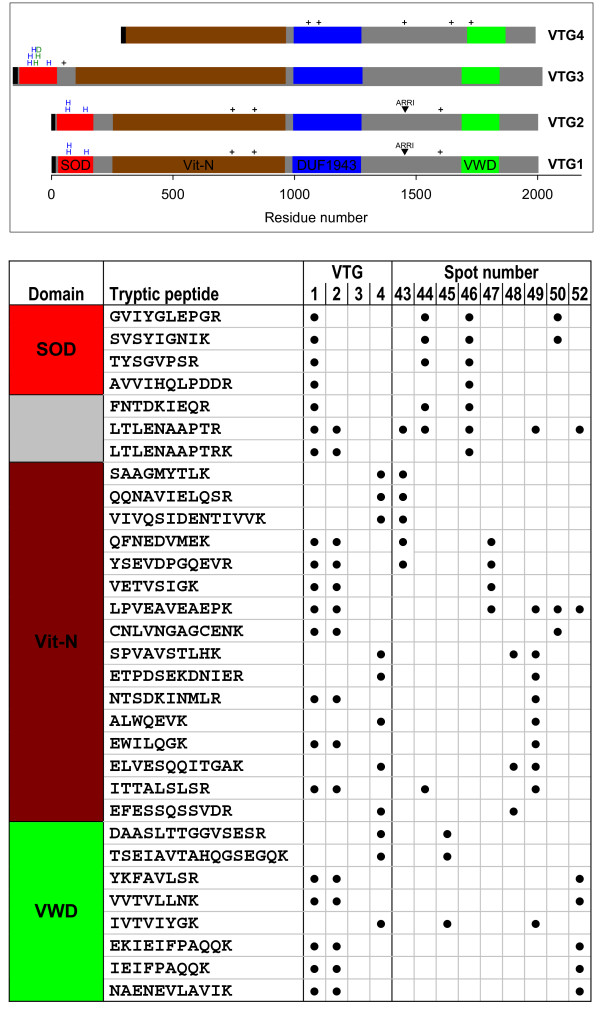
**Assignment of protein spots to the vitellogenins of *Daphnia pulex***. *Daphnia *vitellogenins (VTGs) are generally composed of an N-terminal large-lipid-transfer-module (Vit-N), a domain of unkown function (DUF1943), and a C-terminal von Willebrand-factor type-D domain (VWD). Of the muliple VTGs of *D. pulex*, only four are shown in respect to their domain composition (top). Note that VTG1, VTG2 and VTG3 additionally contain a superoxide dismutase-like domain (SOD) at the N-terminus. Interdomain regions are shown in gray, the signal peptide in black. Conserved residues of the SOD for Cu^2+ ^and Zn^2+ ^binding are indicated by blue (histidines) and green characters (histidines, aspartic acid), respectively. Potential N-linked glycosylation sites are indicated by plus signs. 'ARRI' indicates primary cleavage sites between two arginine residues. The lower part lists the tryptic peptides in the order of their appearance in the VTG sequences and in the analyzed spots.

Most dominant among the identified cold-induced proteins were the vitellogenins (VTGs) and actins. These proteins showed a 4–7-fold induction and were detected in ten spots (43–52). The multiple detection of these proteins and the large discrepancies between the experimental (15–40 kDa) and predicted *M*_r _values (actins: 42 kDa, VTGs: 190–220 kDa) indicate that the main share of the cold-induced protein spots in the lower right diagonal half of the gel (Figure 1C; red-colored spots) were proteolytic cleavage fragments. However, it is important to note that VTG cleavage fragments of 65–155 kDa may naturally occur in developing *Daphnia *embryos (see discussion). The tryptic peptides used for the identification of VTGs covered a large part of the VTG sequences including the superoxide dismutase-like domain (SOD), the large-lipid-transfer module (Vit-N), and the von Willebrand-factor type-D domain (VWD) (Figure 2). None of the tryptic peptides could be allocated to the domain of unkown function (DUF1943) and to the interdomain regions. Based on the high sequence coverage by trypic fragment analysis, two vitellogenins (VTG1, VTG4) could be identified (Figure 2, lower part). Although the present study did not yield any tryptic peptides for the N-terminal SOD-like domain of VTG2, the presence of VTG2 among the analyzed spots cannot be excluded because of the very large sequence identity of VTG2 and VTG1 (98% identity when excluding the SOD-like domain).

**Figure 3:**
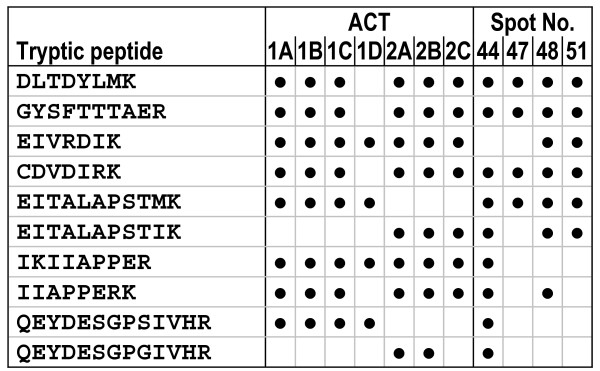
**Assignment of protein spots to the actin sequences of *Daphnia pulex***. The *D. pulex *genome contains seven actin genes which code for cytoplasmic (ACT1A-D) and muscle-specific isoforms (ACT2A-C). The tryptic peptides identified in mass spectrometry are listed in the order of their appearance in the sequence of gene products and gel spots.

Actins were detected in four spots (44, 47, 48, 51). The tryptic peptides used for the identification of actins (Figure 3) covered only the C-terminal half of the 42-kDa proteins, suggesting that the N-terminal half was proteolytically cleaved during the preparation of whole-animal extracts. Proteolytic cleavage is additionally indicated by the discrepancy between experimental (25–36 kDa) and predicted *M*_r _values (42 kDa). Owing to the high sequence identity (≈ 97%), it was impossible to discriminate the expression of cytoplasmic isoforms (ACT1A-D) and muscle-specific isoforms (ACT2A-C). The lower number of tryptic-peptide assignments and the complete lack of EST evidences for ACT1D and ACT2C, however, suggests that these two actins were probably not expressed.

A ten-fold up-regulation in the cold was found for an AAA+ adenosine triphosphatase (ATPase associated with diverse cellular activities; [15]), a fragment of which was detected in spot 42. Additional identifications comprised proteolytic cleavage fragments of a molecular chaperone (HSP90, spot 47), the heavy chain of myosin (MHC-1) and a pyruvate kinase (both in spot 48). Since the latter three proteins were co-identified with actins and VTGs in the same spots, it was impossible to assess their induction states.

Among the remaining identifications was a group of carbohydrate-modifying enzymes with a constitutive or reduced expression in the cold. Constitutive expressions showed the exo-β-1,3-glucanase EXG5 (spot 34) and the α-amylase AMY (spot 35). The cold-repressed proteins included a cellubiohydrolase (CEL7A, spot 1), an endo-β-1,4-glucanase (CEL9A, co-localized with paramyosin in spot 19), an endo-β-1,4-mannanase (MAN5A, co-localized with a β-1,3-glucan-binding protein in spot 30), and the enolase ENO (spot 19).

## Discussion

As a companion study to a previous investigation of acclimatory adjustments of the *Daphnia pulex *proteome to hypoxia [12], the effects of two different acclimation temperatures (10°C and 20°C) on the *Daphnia pulex *proteome were analyzed by 2D gel electrophoresis and mass spectrometry. Temperature acclimation mostly affected the expression of sets of proteins different from those identified under variable oxygen conditions. Several proteins constitutively expressed or subjected to hypoxic induction were also detected in the 2D gels presented here. The specific sets of proteins up- or down-regulated in the cold (10°C) were identified here for the first time.

### Cold-induced protein sets I: Egg yolk proteins and precursors

The most dominant group among the cold-induced proteins in *D. pulex *were the vitellogenins (Table 1). Vitellogenin (VTG) is a precursor of the yolk protein vitellin. It is a lipoglycoprotein that is employed as a vehicle to provide the developing embryo with proteins, lipids, carbohydrates, and other essential resources. In many oviparous animals such as insects and non-mammalian vertebrates, VTG is synthesized in extraovarian tissues (e.g. fat body or liver) and is then transported *via *the blood/hemolymph to the developing oocytes [16,17]. An exception are the decapod crustaceans which show, in addition to the extraovarian synthesis in the hepatopancreas, an intraovarian synthesis of yolk proteins [18]. Since the VTGs of the branchiopod crustacean *Daphnia *spp. are more closely related to insect VTGs than to the yolk protein precursors of decapods [19-21], it is reasonable to postulate a vitellogenic tissue that is homologous to the VTG-synthesizing fat body of insects. Although there are some cytological indications for an endogenous synthesis of yolk proteins in amphigonic oocytes [22], the main site of VTG synthesis in *Daphnia *appears to be the highly polyploid fat cells, which exhibit periodic variations in lipid and glycogen content, cell size and ultrastructure in relation to the parthenogenetic reproduction cycle [22-24].

The screening of the *D. pulex *genome database suggests 19 loci with VTG-like coding sequences. Two gene products, VTG1 and VTG4, were identified in the present study (Figure 2). The additional expression of VTG2, however, which shares a high sequence similarity with VTG1, cannot be excluded. VTG1 and VTG2 are homologous to the vitellogenins DmagVTG1 and DmagVTG2 of *D.magna *[25]. As in *D. magna*, the VTG1 and VTG2 genes are arranged in a tandem array in a back-to-back orientation, which might enable a coordinated hormonal regulation of their transcription [25]. DmagVTG1 and (probably) DmagVTG2 are the most abundant polypeptides in *D.magna *parthenogenetic eggs at initial stages of development [19]. At least one of the primary cleavage sites is present in VTG1 and VTG2 of *D. pulex *(Figure 2, top: 'ARRI'). Given the high sequence identity (88–90%) between the corresponding VTGs of both *Daphnia *species, it is likely that primary cleavage fragments of similar size occur in the developing eggs of *D. pulex *as well. However, none of these primary cleavage fragments could be detected in full length (65–155 kDa) among the analyzed spots, which contained only smaller VTG fragments of 18–27 kDa, possibly as a consequence of a residual proteolytic activity during the preparation of whole-animal extracts. Alternatively, smaller-than-expected fragments may have arisen prior to extract preparation by an advanced cleavage of yolk material during embryonic development.

The 4–7-fold up-regulation of VTGs in 10°C acclimated *D. pulex *(Table 1) was an unexpected finding. About 50–100 adult daphnids were randomly sampled irrespective of their reproductive states for single protein extractions. The protein extracts consequently contained contributions from parthenogenetic eggs and embryos in the brood chamber as well as from maternal tissues. A greater share of vitellogenin in the protein extracts from 10°C acclimated animals may therefore result from a greater amount of eggs in the ovaries and the brood pouch or from an increased vitellogenin concentration in the synthesizing tissues, ovaries, eggs and embryos. An inspection of both acclimation groups did not reveal any differences in clutch size or in the share of animals carrying eggs and embryos. Previous findings on the impact of temperature on clutch sizes in *Daphnia *are ambiguous: there were reports on lowered [9], unchanged [11] or increased [26] clutch sizes in *D. magna *at lower temperatures. In this study, the protein concentration in the extracts was quantified and the extracts were appropriately diluted to guarantee the application of identical amounts of protein (142 μg protein) per 2D gel. Compared to the extracts from 20°C acclimated animals, the extracts from 10°C acclimated animals had a 50% higher protein concentration. The slower growth and development of *D. pulex *in the cold may possibly result in a higher concentration of whole-body protein with the VTGs particularly contributing to this effect.

A striking feature of VTG1-VTG3 is the presence of an N-terminal superoxide dismutase (SOD)-like domain (Figure 2), which is related to the Cu/Zn SODs of prokaryotes [25]. The catalytic activity of this class of SODs depends on Zn^2+ ^and Cu^2+ ^ions, which are coordinated by six histidine residues and one aspartic residue [27]. These residues are still present in VTG3. VTG1 and VTG2 have lost all Zn-binding residues and one of the four histidine residues involved in Cu^2+ ^binding. Functional studies on the purified yolk-protein complexes of *D. magna *revealed some residual SOD activity per constituent VTG chain (≈ 1%, in comparison to the activity of a bovine Cu/Zn SOD) [19]. Because of the great number of VTG loci in the *D. pulex *genome and the presence of an apparently intact SOD-like domain in VTG3 (for which EST evidence is available), it is difficult to analyze any (residual) detoxifying capacity of VTG1 and VTG2. Future experimental studies will be necessary to evaluate the suggested implications of the SOD-like domains of the *Daphnia *VTGs in superoxide detoxification [19] and copper binding/transportation [25].

### Cold-induced protein sets II: Cytoskeleton and muscle proteins

Actins were the second large set of proteins up-regulated in the cold (Table 1). Although actins were often co-identified with VTGs during the proteomic analysis, the identification of only actin in spot 51 indicates the manifold induction of these proteins. Actin is a highly conserved protein. As major building block of the cytoskeleton and the thin filaments of myofibrils, it is involved in many important cell functions including cell motility, muscle contraction and intracellular transport. Actin generally occurs in multiple isoforms which are expressed in a tissue- and development-specific manner [28]. Compared to the genomes of human, mouse, and fly, which contain six actin loci [29], seven actin loci are present in the genome of *D. pulex *(Figure 3). Four of the predicted amino acid sequences (ACT1A, ACT1B, ACT1C, ACT1D) of *D. pulex *are related to cytoplasmic actin isoforms (5C, 42A) of *Drosophila melanogaster *[28,30] The other three *D. pulex *sequences (ACT2A, ACT2B, ACT2C) are similar to the muscle-specific actin isoforms (57B, 79B, 87E, 88F) of *Drosophila*. The ACT2C gene is very likely a pseudogene as it lacks about 50% of the actin sequence information. Among the putative cytoplasmic actins of *D.pulex*, ACT1D possesses less conserved sequence characteristics. The complete lack of EST support for ACT1D and ACT2C suggests that only three cytoplasmic and two muscle-specific actin isoforms are expressed in *D. pulex*. Because of high sequence identity, a discrimination between these isoforms was not possible in the present study.

**Figure 4:**
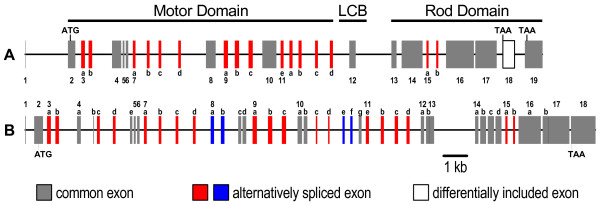
**Myosin genes of *Drosophila melanogaster *and *Daphnia pulex***. (A) The myosin heavy chain (MHC) gene of *D. melanogaster *(FlyBase annotation ID:CG17927) showing the common and alternatively spliced exons (LCB, light chain-binding domain) [31,32,58] (B) Putative architecture of the muscle MHC gene of *D. pulex *(scaffold_6: 2047569–2071828). ATG and TAA indicate the start of translation and the stop codon, respectively. In the *Drosophila *MHC transcripts, the sequence of the terminal exon can be replaced by that of the 'differentially included exon'.

Two additional muscle proteins, the heavy chain(s) of muscle myosin (e.g. MHC-1) and a paramyosin (PMY), were identified on the 2D gels (Table 1). These proteins were detected in separate spots together with other proteins, which make an assessment of induction state difficult. The MHC gene of *D. pulex *deserves special attention as it shares interesting features with the MHC gene of *Drosophila melanogaster *(Figure 4) [58]. In contrast to many complex organisms with physiologically distinct muscle types, in which MHC isoforms are encoded by multiple genes, at least 15 muscle MHC isoforms are expressed in *Drosophila *from a single-copy gene by alternative splicing. Many of these isoforms show tissue- or development-specific expression [29,31,32] The *D. pulex *genome also contains a single-copy muscle MHC gene, whose exon structure shows similarity to the MHC gene structure of *Drosophila*. Given the complexity of the MHC gene and the, at present, only scarcely available transcript information, no conclusions can be drawn about the number and identity of MHC isoforms in *D. pulex*.

In general, a reduction of ambient temperature is immediately responded by a decrease of muscular performance in *Daphnia*. For example, the limb beating rate decreases which in turn reduces the uptake of oxygen and food. Likewise, the heart rate decreases with the consequence of a reduced hemolymph transport of substrates [3,5,8,11]. However, the heart and limb beating rates were frequently not much different in *Daphnia *species at identical ambient and acclimation temperatures of 10–12°C or 18–20°C [3,5]. In addition, maximum swimming activity of 10°C acclimated *D. magna *was found to be similar to that of 20°C acclimated animals [6]. In poikilothermic animals, the concentration of enzymes involved in cellular metabolism frequently increases with decreasing acclimation temperatures to prevent a too strong depression of metabolic rate (metabolic cold adaptation) [7]. Such a type of long-term compensatory control may apply as well to the cytoskeletal or muscular proteins to maintain a similar level of muscular performance at lower acclimation temperatures.

### Cold-repressed protein sets: Proteolytic enzymes

In the cold, different classes of enzymes mainly involved in extracellular digestion were down-regulated. In other words, the capacity for the digestion of proteins increased with acclimation temperature (Table 1). The identification comprised serine peptidases of the chymotrypsin family S1, metallo peptidases of the astacin/adamalysin family M12, and the carboxypeptidase A family M14 (classification according to the MEROPS database) [33]. A screening of the *D. pulex *genome database revealed more than 200 loci with coding sequences for serine-peptidase domains, 36 loci with astacin-like coding sequences, and 30 loci coding for carboxypeptidase-like domains. However, not all predicted gene products are involved in digestive processes. Serine proteases of the chymotrypsin family, for example, are involved in multiple physiological functions such as digestion, degradation, blood clotting, immunity, and development [34]. Nevertheless, the sheer number of peptidase genes in the *D. pulex *genome indicates large-scaled gene family expansions that might reflect specific adaptations to the lifestyle of a planktonic filter feeder in a highly variable aquatic environment [35].

**Figure 5:**
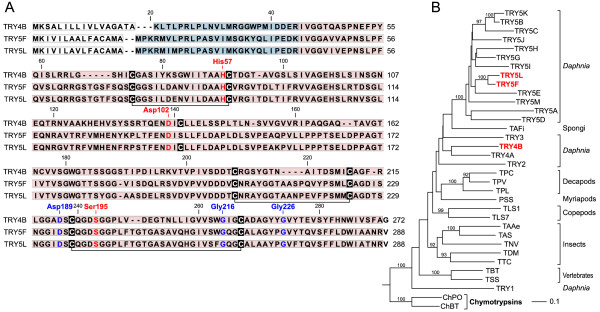
**Trypsin-like proteins of *Daphnia pulex***. (A) Derived amino-acid sequence and domain structure of three trypsin genes (TRY4B, TRY5F, and TRY5L) from *D. pulex*. Predicted domain characteristics include the N-terminal signal peptide (white frame), the propeptide (blue), the chymotrypsin-like domain (red), the conserved disulfide bridges (connected cysteine residues), the catalytic triade (red characters), and substrate-specificity residues (blue characters). Residues numbering was taken from bovine chymotrypsinogen [59]. (B) Phylogenetic tree for selected trypsin-like sequences based on a multiple-sequence alignment of the trypsin-like domain including three adjacent propeptide residues (see Additional file 1). Proteins detected in the present study are labeled in red. The tree was constructed using the neighbor-joining algorithm and was rooted with chymotrypsin sequences. Bootstrap analysis was performed with 100 replicates (boostrap values <80 are omitted). Abbreviations and NCBI accession numbers: TRY1-TRY5M, *Daphnia pulex*; TAFi, trypsin from *Aplysina fistularis *(AAO12215); TPC, trypsin from *Paralithodes camtschaticus *(AAL67442); TPV, trypsin from *Litopenaeus vannamei *(CAA75311); TPL, trypsin from *Pacifastacus leniusculus *(CAA10915); PSS, plasminogen activator from *Scolopendra subspinipes *(AAD00320); TLS1 and TLS7, trypsin from *Lepeophtheirus salmonis *(CAH61270, AAP55755); TAAe, trypsin from *Aedes aegypti *(P29787); TAS, trypsin from *Anopheles stephensi *(AAB66878); TNV, trypsin from *Nasonia vitripennis *(XP_001599779); TDM, trypsin from *Drosophila melanogaster *(P04814); TTC, trypsin from *Tribolium castaneum *(XP_967332); TBT, trypsin precursor from *Bos taurus *(Q29463); TSS, trypsin-1 precursor from *Salmo salar *(P35031); ChPO, chymotrypsinogen 2 from *Paralichthys olivaceus *(Q9W7Q3); ChBT, chymotrypsinogen A from *Bos taurus *(P00766).

**Figure 6:**
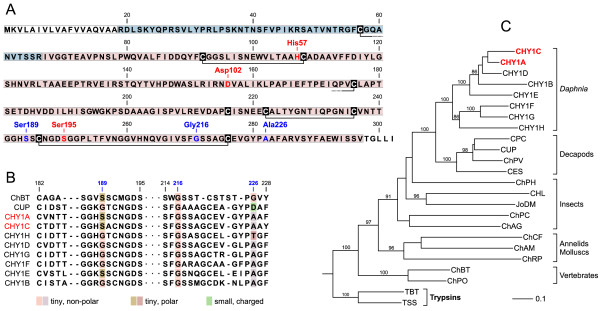
**Chymotrypsin-like proteins of *Daphnia pulex***. (A) Derived amino-acid sequence and domain structure of the CHY1A gene from *D. pulex*. Predicted domain characteristics include the N-terminal signal peptide (white frame), the propeptide (blue), the chymotrypsin-like domain (red), the conserved disulfide bridges (connected cysteine residues), the catalytic triade (red characters), and substrate-specificity residues (blue characters). (B) Sequence alignment of chymotrypsin-like enzymes showing the substrate recognition site with the primary specificity (S1) residues at 189, 216 and 226 (numbering system of bovine chymotrypsinogen; [59]). The shape (tiny, small) and electrostatic character (non-polar, polar, charged) of S1 residues is indicated by color shading. (C) Phylogenetic tree for selected chymotrypsin-like sequences based on a multiple-sequence alignment of the chymotrypsin-like domain including four adjacent propeptide residues (see Additional file 2). Proteins detected in the present study are labeled in red (CHY1A and CHY1C). The tree was constructed using the neighbor-joining algorithm and was rooted with trypsin sequences. Bootstrap analysis was performed with 100 replicates (boostrap values <80 are omitted). Abbreviations and NCBI accession numbers: CHY1A-H, *Daphnia pulex*; CPC, collagenolytic protease from *Paralithodes camtschaticus *(AAL67441); CUP, collagenolytic protease from *Celuca pugilator *(P00771); ChPV, chymotrypsin BII from *Litopenaeus vannamei *(CAA71673); CES, protease from *Euphausia superba *[39]; ChPH, protease from *Pediculus humanus corporis *(AAV68346); CHL, collagenase precursor from *Hypoderma lineatum *(P08897); JoDM, Jonah 43E from *Drosophila melanogaster *(NP_724699); ChPC, chymotrypsin precursor from *Phaedon cochleariae *(O97398); ChAG, protease from *Anopheles gambiae *(AGAP005663-PA); ChCF, protease from *Chlamys farreri *(ABB89132); ChAM, chymotrypsinogen from *Arenicola marina *(CAA64472); ChRP, serine peptidase 2 from *Radix peregra *(ABL67951); ChBT, chymotrypsinogen A from *Bos taurus *(P00766); ChPO, chymotrypsinogen 2 from *Paralichthys olivaceus *(Q9W7Q3); TBT, trypsin precursor from *Bos taurus *(Q29463); TSS, trypsin-1 precursor from *Salmo salar *(P35031).

The identified serine peptidases comprised three trypsin-like proteins (TRY4B, TRY5F, TRY5L) and two chymotrypsin-like proteins (CHY1A, CHY1C). The presence of N-terminal signal and propeptide sequences classifies these candidates as secretory proteins that are synthesized as inactive pro-enzymes (zymogens). All sequences contain the characteristic residues of the catalytic triade (His57, Asp102, Ser195; Figures 5A and 6A) [59]. Substrate specificity is usually determined by three residues at the S1 site which is a pocket adjacent to Ser195 [36]. The S1-site residues of trypsin are Asp189, Gly216 and Gly226 [37]. All three residues are present in the detected trypsins of *D. pulex *(Figure 5A). Multiple-sequence alignment (Additional Files 1, 2) and phylogenetic-tree analysis of serine peptidase sequences from the *D. pulex *genome database revealed many other trypsin-like proteins. Two of them (TRY5F, TRY5L) together with 11 other sequences from *D.pulex *form a monophyletic cluster (Figure 5B). In CHY1A and CHY1C, the primary specificity residues comprise Ser189, Gly216 and Ala226 (Figure 6B). The first two residues are the same as in bovine chymotrypsin [37]. At the third position, Ala226 replaces the typical Gly226. These two residues are similar in shape and electrostatic character, suggesting that substrate specificity is not significantly altered by this replacement. CHY1A and CHY1C together with six additional chymotrypsin-like proteins from *D. pulex *form a monophyletic cluster (Figure 6C). *D. pulex *chymotrypsins are closely related to the C-type brachyurins (MEROPS classification: S01.122), which include the decapod chymotrypsins and collagenolytic proteases [38-42] C-type brachyurins are characterized by a broad substrate specificity [41]. Among the *D.pulex *chymotrypsins, an even enlarged range of substrate specificity may be assumed because of the sporadic replacements of Ser189 and Gly226 by residues of different electrostatic properties (Figure 6B).

The MS analysis could identify and assign only those tryptic peptides which were specific for mature proteolytic enzymes. No support was obtained for the N-terminal signal peptides, which direct the nascent proteins to the secretory pathway, and for the pro-peptides, which shield the active sites in the immature trypsinogens or chymotrypsinogens (Figure 5 and 6). Therefore, it can be assumed that the proteases originated from the gut lumen, which (in *D. magna*) contain the major share of proteases [43]. During the preparation of whole-animal extracts for the present study, intestinal proteins such as proteases are included along with those from other tissues. The presence of high amounts of proteases causes methodical problems [12], resulting in a contribution of proteolytic fragments to the observed protein spots. On the other hand, the high concentration of proteases being present in the whole-animal extracts documents a high digestive capacity for nutritional protein resources which increases with acclimation temperature. The marked induction of proteases between 10°C and 20°C acclimated animals probably reflects a higher rate of protein turnover at the higher temperature. Between identical ambient and acclimation temperatures of 10 and 20°C, the oxygen consumption rate of *D. magna *increased by 30% [4] and that of *D. pulex *by 60% (unpublished results). Accordingly, the observed induction of proteolytic capacity by a factor of 2–5 (Table 1: trypsin, chymotrypsin) may reflect at least in parts the temperature effect on metabolic rate in acclimated *D. pulex*. In addition, higher needs for proteins may arise at higher temperatures due to modifications in the allocation and/or requirement of nutrient resources (e.g. enlarged protein needs for growth and reproduction). Previous reports on the impact of temperature on clutch sizes in *Daphnia *were ambiguous; however, a reduction of vitellogenin and protein concentration was detected in this study between 10°C and 20°C acclimation (see Discussion above). At 20°C acclimation (in comparison to 10°C acclimation), the higher growth rate (and possibly a higher reproduction rate) of *D. pulex *and/or a faster passage of nutrients through the digestive tract with possibly incomplete nutrient digestion and reduced assimilation efficiency goes hand in hand with a reduced concentration of total protein and vitellogenin in the animals. These relationships at least indicate higher demands for proteins at 20°C acclimation, which may explain the induction of intestinal proteases.

### Miscellaneous proteins

Among the miscellaneous proteins with an unambiguous (one spot-one protein) identification were several carbohydrate-modifying enzymes, which were either down-regulated in the cold (cellubiohydrolase, enolase) or remained constitutively expressed (α-amylase, exo-β-1,3-glucanase), and an AAA+ ATPase, which was strongly up-regulated under cold conditions. AAA+ ATPases are molecular machines that are involved in a variety of cellular functions including vesicle transport, organelle assembly, membrane dynamics and protein unfolding [15]. They contribute to the non-destructive recycling of proteins, play an important role in protein quality control (e.g. chaperone function), and can act as microtubule motor proteins or microtubule-severing enzymes [15].

## Conclusion

Major sets of proteins (egg yolk proteins and precursors, cytoskeleton and muscle proteins, proteolytic enzymes) were differentially expressed in 10°C and 20°C acclimated *D. pulex*. Compared to 10°C, the acclimation to 20°C was associated with a decrease of vitellogenins, actins and even total protein concentration, as well as with an increase of proteases. The increase of proteolytic enzymes probably reflects a higher cellular demand for amino acids, which may result from higher growth and reproduction rates and/or from a lower efficiency of intestinal protein digestion/assimilation. The decrease of protein reserves (vitellogenins, actins or total protein) also indicates an increasing bottle-neck in the amino acid supply of cells. Conversely, the acclimation to cold conditions induced an increase in protein concentration which may be related to metabolic cold adaptation, a phenomenon for which multiple physiological support exists. Metabolic cold adaptation is a compensatory mechanism which ensures a relative constancy of metabolic rate and muscular performance. Particularly, the increase of actins in the cold maybe related to a compensatory control of muscular proteins to establish a relative constancy of muscular activity and performance.

## Methods

### Acclimation conditions

Water fleas, *Daphnia pulex*, were raised in the laboratory as described previously [12]. The animals were acclimated at least for three weeks (mostly months) to 10°C or 20°C at normoxic conditions (100% air saturation; oxygen partial pressure: 20 kPa), which was obtained by mild aeration using an aquarium pump. To guarantee an adequate nutrient supply at each acclimation temperature, animals were fed with green algae (*Desmodesmus subspicatus*) *ad libitum *(>1 mg C L^-1^) every second day. Only adult females were used for protein extraction.

### Proteomics

Protein extraction, two-dimensional gel electrophoresis and statistical analysis of protein expression were carried out as described previously [12]. Spots showing a sufficient size and staining intensity (relative spot volume, *V*_rel _> 0.1%) and differential expression between 10°C or 20°C acclimation, were excised from representative gels and subjected to in-gel digestion using trypsin and mass spectrometric analysis (nano-HPLC-ESI-MS/MS) [12]. Ratios of relative spot volumes at both temperatures were considered as induction factors. Several spots of high but constitutive expression were also included in the analysis.

### Identification and characterization of proteins

Proteins were identified by correlating the ESI-MS/MS spectra with the "Frozen Gene Catalog" of the *D. pulex *v1.1 gene builds (July, 2007) [2] using the MOWSE-algorithm as implemented in the MS search engine MASCOT (Matrix Science Ltd., London, UK) [44]. The "Frozen Gene Catalog" contains all manual curations as of July 3, 2007 as well as automatically annotated models chosen from the "Filtered Models" v1.1 set. "Filtered Models" is the filtered set of models representing the best gene model for each locus. The putative function of identified proteins was inferred by sequence homology either from the automated blastp search provided by Joint Genome Institute [2] or from a manual blastp search provided by NCBI. Derived protein sequences were checked for the presence of N-terminal signal sequences using the SignalP V3.0 server [45-47]. The theoretical molecular weight (*M*_r_) and isolectric point (pI) of mature proteins (without N-terminal signal peptide) was calculated using the ExPASy Proteomics tool "Compute pI/MW" [48-50]. Characteristic domains of protein families were identified using the conserved domain database (CDD) and search engine v2.13 at NCBI [51,52]. Putative N-glycosylation sites in vitellogenins were predicted using the NetNGlyc 1.0 Server [53].

### Sequence alignments and phylogenetic analysis

Multiple-sequence alignments were performed using the T-Coffee algorithm [54-56]. Phylogenetic trees were constructed using the neighbor-joining algorithm [57] and a bootstrap analysis with 100 replicates.

## Abbreviations

*M*_r_: molecular weight; pI: isolectric point; *V*_rel_: relative spot volume.

## Authors' contributions

SS and MK were involved in the culturing of animals and performed the protein extraction as well as the 2D-PAGE. 2D-gel image analysis was carried out by TL, BZ and RP. JM and CF were responsible for mass spectrometry and protein identification. RP and SS retrieved the information contained in Table 1. Figures were designed by RP. The annotation and manual curation of identified genes, the sequence alignments and phylogenetic analysis were performed by RP. BZ, RJP, RP and SS conceived and coordinated the study, and prepared the manuscript.

## Supplementary Material

Additional File 1 **Multiple sequence alignment of trypsin-like sequences**. The multiple-sequence alignment was performed using the T-Coffee algorithm [54]. NCBI accession numbers for the symbolic sequence names are listed in the Figure legend 5. Click here for file

Additional File 2 **Multiple sequence alignment of chymotrypsin-like sequences**. The multiple-sequence alignment was performed using the T-Coffee algorithm [54]. NCBI accession numbers for the symbolic sequence names are listed in the Figure legend 6. Click here for file
