# Supplementary material for: Acclimatory responses of the Daphnia pulex proteome to environmental changes. II. Chronic exposure to different temperatures (10 and 20°C) mainly affects protein metabolism
Source: BMC Physiol. 2009 Apr 21;9:8. doi: 10.1186/1472-6793-9-8 (PMC2678069; doi:10.1186/1472-6793-9-8)
Supplement: Additional File 1 — Multiple sequence alignment of trypsin-like sequences. The multiple-sequence alignment was performed using the T-Coffee algorithm [54]. NCBI accession numbers for the symbolic sequence names are listed in the Figure legend 5. [file 1472-6793-9-8-S1.pdf]

```
T-COFFEE, Version_5.49Wed Dec 19 12:12:01 2007
   Cedric Notredame
   CPU TIME:34 sec.
   SCORE=56
   *
    BAD AVG GOOD
   *
   PSS      :   57
   TAAe     :   54
   TAFi     :   55
   TAS      :   51
   TBT      :   55
   TSS      :   57
   TDM      :   50
   TLS1     :   53
   TLS7     :   54
   TNV      :   53
   TPC      :   57
   TPV      :   58
   TPL      :   58
   TRY1     :   46
   TRY2     :   53
   TRY3     :   53
   TRY4A    :   57
   TRY4B    :   55
   TRY5A    :   55
   TRY5B    :   59
   TRY5K    :   58
   TRY5C    :   58
   TRY5D    :   56
   TRY5E    :   56
   TRY5F    :   59
   TRY5L    :   59
   TRY5G    :   59
   TRY5I    :   58
   TRY5H    :   57
   TRY5J    :   59
   TRY5M    :   56
   TTC      :   53
   ChBT     :   57
   ChPO     :   54
   cons     :   56
```

```
PSS      -M-NS-F-TIL-IV---TYFSLAF----GS-RCGI-------------------
TAAe     ------------------------------------------------------
TAFi     --------VRA-TLVC---LAAAV----SGDNYV--LPRLPLSFMLR--RGVN-
TAS      MSNKI--AILLAILVAVVACVEVQ----ASQRPR---TARPLPHFLP---RPQ-
TBT      --MHP--LLIL-AF---VGAAVAF----PS------------------------
TSS      --MIS--LVFV-LL---IGAAFA-------------------------------
TDM      -MLKI--VILL-SA---VVCALGG----TVPEGL--LPQL--------------
TLS1     --MNK-FISALFFL---VATALAK----PN-DGI--TKSIMDM-----RLMYP-
TLS7     --RFF--CAL-LFL---VVAANAV----PPQ------IKYSESFMRV--KSMR-
TNV      -------MLICN----AFCYVAAMTAEMQLEQAC--RPNIVIAVQTL--AKFSL
TPC      --MKY--LVFC-LL---LGAAFAA----PSR-KP--TFRRG-------------
TPV      ----S--LVLC-LL---LAGAFAA----PSR-KP--TFRRG-------------
TPL      PAMKT--LVFC-LL---LVGALAA----PSRRL-----RFPP------------
TRY1     -MCRF--LFIF-LL---WNYATRV----DTA----------------SIADAP-
TRY2     --MKI--YLVLLT---VFTVTTTA--------SS--LARFPLSVFLD--TELI-
TRY3     -MAKLVAVIVGAIC---VQCFFAL----PS---------PGRNDLVWR------
TRY4A    --MKF--LILVCC---LLSCAAG--------NSL--FPRLPL-FYRT--DQPS-
TRY4B    -MKSA--LILLIV---LVAGATA--------KLT--LPRLPLNVLMR--GGWP-
TRY5A    -MMKH--SILV-TL---FACVLAV-GAAPS------------------------
TRY5B    -MNKI--ILLA-AL---FACAYAA----PQ-RMV--LPRLPAKVILN--GKYQ-
TRY5K    -MNKI--ILLA-AL---FACAHAA----PH-HMV--LPRPPANVILN--GQYQ-
TRY5C    -MNKI--VILA-LL--YFACIHAV----PQ-RMV--LPRLPASVILS--GKYQ-
TRY5D    --MKL--IILA-TF---VACAFGA----PH-RMV--LPRLPVSVILT--GKFQ-
TRY5E    --MKI--IVLA-AF---FAFALAM----PK-RMV--LPRLPAAVMMS--GKYQ-
TRY5F    --MKI--VILA-AL---FACAMAM----PK-RMV--LPRLPASVIMS--GKYQ-
TRY5L    --MKI--VILA-VL---FACAMAM----PK-RMI--MPRLPASVIMK--GKYQ-
TRY5G    --MKF--LVLA-AL---FACACAL----PQ-RMV--LPRLPASVISK--GKFQ-
TRY5I    --MKF--VVLA-TL---LTYSWAA----PH-PPI--LPRLPIKDIVS--G-HQ-
TRY5H    --MKC--FLLA-TL---LVFAWAA----PQQRAV--LPRLPLNIILN--KRFQ-
TRY5J    --MKF--VILAATI---LACVIAN----PSTRSV--LPRLPLKVLLS--GKFQ-
TRY5M    --MKT--VVLLATF---VVCCSTV----IS-ESVTSSPHRSASLIMNNRGSYQ-
TTC      --MKS--FLLIVFL---VATASAV----PS-YL----------------RTNS-
ChBT     -----------------------CGVP---------------------------
ChPO     --MGI--LSCL-AF---AGAAYGCGSP---------------------------

cons
```

```
PSS     -------------------------------------------KNGPMLDEFNR
TAAe    -------------------------------------------TMVERSDSSGR
TAFi    ------------------------------------------------LVPESK
TAS     ------------------------------------------------YSVGHR
TBT     -------------------------------------------------DDDDK
TSS     -------------------------------------------------TEDDK
TDM     ---------------------------------------------------DGR
TLS1    ------------------------------------------------NQFDGR
TLS7    ------------------------------------------------HRFGGR
TNV     QIKISVFEADTCGVLANCLTFFYYTLIFLNFVLIFAIIARTVALGVSDNPDDGR
TPC     ---------------------------------------------------LNK
TPV     ---------------------------------------------------LNK
TPL     --------------------------------------------------NNYK
TRY1    ------------------------------------------------ALRPTR
TRY2    -----------------------------------------------RTKRDGR
TRY3    -----------------------------------------------RKAATPL
TRY4A   ------------------------------------------------LLSGEK
TRY4B   ------------------------------------------------MIDDER
TRY5A   --------------------------------------------------RDNK
TRY5B   ------------------------------------------------LIPEDK
TRY5K   ------------------------------------------------LIPEDK
TRY5C   ------------------------------------------------LIPEDK
TRY5D   ------------------------------------------------FVPEDK
TRY5E   ------------------------------------------------LIPKDK
TRY5F   ------------------------------------------------LIPEDK
TRY5L   ------------------------------------------------LIPEDR
TRY5G   ------------------------------------------------LIPSNK
TRY5I   ------------------------------------------------LKLSEK
TRY5H   -----------------------------------------------NAISSDR
TRY5J   ------------------------------------------------MIPEDK
TRY5M   ------------------------------------------------RIPQEK
TTC     ------------------------------------------------LLPDGR
ChBT    -------------------------------------------AIQPVLSGLSR
ChPO    -------------------------------------------AIPPVITGYSR

cons
```

```
PSS      IVGGEAAEPGEFPWQISLQVVS-W------YG--SYHYCGGSILDESWVVTAAH
TAAe     IVGGFEVPVEEVPFQVSLSGVG------------SSHFCGGSLLSERWVMTAGH
TAFi     IVGGDPVNKGDVPWQVSLQREG-F------FG--RSHFCGGSILDADTVLTAAH
TAS      IVGGFEIDVSETPYQVSLQYF-------------RSHRCGGSVLNNKWVLTAAH
TBT      IVGGYTCAENSVPYQVSLNA--------------GYHFCGGSLINDQWVVSAAH
TSS      IVGGYECKAYSQTHQVSLNS--------------GYHFCGGSLVNENWVVSAAH
TDM      IVGGSATTISSFPWQISLQRSG-------------SHSCGGSIYSANIIVTAAH
TLS1     IVGGEEAEPNAFPYQLSLRSGG-L--------L-SYHFCGASIYDEKTAITAAH
TLS7     IVGGEEVEPNSIPFQVSFQTTG------------GFHFCGASVMDKDTIITAAH
TNV      VVGGYETSIEQHPYQVSLRYKG-------------RHKCGGAIIAEDWVITAAH
TPC      IVGGTEVTPGEIPYQLSFQDTS-F-------GG-EFHFCGASIYKDTWAICAGH
TPV      IVGGSDATPGELPYQLSFQDVS-F-------GF-AFHFCGASIYNENWAICAGH
TPL      IVGGTDASLGEFPYQLSFQEKF-L-------GF-SFHFCGASIYNENYAITAGH
TRY1     IVNGEEVRDGEVTWQISLQLSS-AVSGENRQRT-MSHFCGGAIINSQWAITAAH
TRY2     IIGGADAKEGEFPWMVSLQRNG-F------FG--RSHFCAGSIADARSIITAAH
TRY3     IVGGEEALPNEFPFMVSMQSLA-V------GGT-YNHRCGGAVINERWVVSASH
TRY4A    IVGGTQATPNEFPWQISLQRRG-V------LSS-YSHSCGGSVYNNNYIIDASH
TRY4B    IVGGTQASPNEFPYQISLRRLG-------------SHICGASIYKSGWIITAAH
TRY5A    IINGGEVVPYSLPFVVTIQTKT--------GNT-FTHLCAGSILDESTILTSAG
TRY5B    IVGGSEVVPNSLPFQISLQRRS--------FGG-FSQSCGGSILNENTILDAAH
TRY5K    IVGGSEVVPNSLPFQISLQRRS-S------FGG-FSHSCGGSILNENTILNAAH
TRY5C    IVGGKEVVPNSLPFQISLQRKS-A------SGI-FSQSCGGSILNENTILDAAH
TRY5D    IVGGAEVVPNSLPFVVSLQRKF-L------T-L-STHACAGTILDEKTILTAAH
TRY5E    IVGGAVVEPNSLPFQVSLQRLSGT------TGQ-YSQSCGGSILDDSTILNAAH
TRY5F    IVGGAVVAPNSLPFQVSLQRRG-S------TGS-FSQSCGGSILDANVIIDAAH
TRY5L    IVGGVIVEPNSLPFQVSLQRRG-S------TGT-FSQSCGGSILDENVILDAAH
TRY5G    IVGGTEVEPNSLPFQVSLQRKG-L------TGL-YSQSCGGSILDENTILDAAH
TRY5I    IIGGVEVVPNSLPFQVSIQRGS--------AGV-YTQSCGGSILNETTVLTAAH
TRY5H    ILSGSEVVPNSLPFQVSLQRRG-L------NGA-YVQVGGGSILDDSTILHAAH
TRY5J    IVGGTEVAPNSLPFQISLQRRG-LL----PNSA-YSHICGGSILDATTILDAAH
TRY5M    IVGGTIVVPNSLPFQVSIQRRS-D------AGSRWAHYCGGVILDANVVITAAH
TTC      IIGGSSISISSVPWQVSLQYYG-------------SHICGGSIISAKYIVTAAH
ChBT     IVNGEEAVPGSWPWQVSLQDKT------------GFHFCGGSLINENWVVTAAH
ChPO     IVNGEEALPHSWPWQVSLQDYT------------GFHFCGGSLINENWVVTAAH

cons     ::.*        .  :::                  :  .. :      : :.
```

```
PSS      CVEGM---NPSDLRILAGEHNFKKEDGTEQWQDVIDIIMHKDYVYSTLENDIAL
TAAe     CAASG----QTNLQVRIGSSQ--HASG-GQLIKVKKVNRHPKYDEVTTDYDFAL
TAFi     CTDGQ---VPSGITVVAGDHVLSTTDGDEQVVGVASISEHPEYNSRTFYNDICV
TAS      CTDGL---DASSLSVRLGSSK--HASG-GTVVRVARIVEHPKYDGNTIDHDYCL
TBT      CYQ-------YHIQVRLGEYNIDVLEGGEQFIDASKIIRHPKYSSWTLDNDILL
TSS      CYK-------SRVEVRLGEHNIKVTEGSEQFISSSRVIRHPNYSSYNIDNDIML
TDM      CLQSV---SASVLQVRAGSTYWSS---GGVVAKVSSFKNHEGYNANTMVNDIAV
TLS1     CCQNL----PKYAKVVAGDHSQHSVSGFEQKIRVKSYVIHPDFGTSGVNNDICI
TLS7     CCDSF---FPWEVQVVAGEHDLFSTSGDEQTIDVSDITYHEMFASHGTNYDVCL
TNV      CLKSS---NPSHLSIKAGSSTL---GGRGQVVDVHHVIRHEDYSRRESDYDIAL
TPC      CVQGEDFDSPASLQIVAGDHTLYSAEGNEQKIAVSKIIQHEDYNGFSISNDISL
TPV      CVQGEDMNNPDYLQVVAGEHNRDVDEGNEQTVVLSKIIQHEDYNGFTISNDISL
TPL      CVYGDDYDNPSGLNIVAGELDMSVNEGSEQTIAVSKIILHENLDYDLLDNDISL
TRY1     CVYQK--MYITSDNLFVVFNTTNMKLPDAANIGVEK-IFSANYNEDTKENDLAL
TRY2     CLEEL--H-PIGVWAVAGEHRLDLVSGFEQELRAAQFVLHEEYDPDYLRNDIGI
TRY3     CTENA--L-VGATRIAAGEHSLSTTSGFEQFSDVEMFVMHPNYDTVTLENDIVL
TRY4A    CVDGV--TDVGTLRVVAGEHSLTQTSGYEQIRSVLSYKMHESYNSNTFANDIAL
TRY4B    CTDGT---AVGSLSIVAGEHSLSINSGNEQTRNVAAKHEHVSYSSRTQENDICL
TRY5A    CVANQ--A-TRNLQVVASEHSLGTISGEEQNRLVASILPNAAYDANTNERDIAI
TRY5B    CVDGV--TDLTIFRVVAGEHSLSVVSGLEQNRDVSGYLMHPDYDTSTSSNDIAL
TRY5K    CVVGI--TDLTIFRVVAGEHSLSVVSGLEQNRDVSEYLMHPDYVQRTFFNDIAL
TRY5C    CVDGV--ADVSIFRVVAGEHDLFQVSGLEQNMDVNNILMHPDYNKKTSENDIAL
TRY5D    CLDY---DNATIFRVVAGEHSFLNESGMEHNSNVDSYLMHPDYSPITLENDIAL
TRY5E    CVRGV--D-PASMLIVAGEHSLSQESGLEQNRFVINTTVHEKYDFLTYENDISL
TRY5F    CVRGV--TDLTIFRVVAGEHSLRTDSGLEQNRAVTRFVMHENYKPLTFENDISL
TRY5L    CVRGI--TDLTIFRVVAGEHSLRNVSGLEQNRGVTRFVMHENFRPSTFENDICL
TRY5G    CVDGA---NIPNLRIVAGEHSLSVESGLEQNRLVTRVATHPRYNSATYEDDISL
TRY5I    CVDGA---VIRALRVVAGEHSLNDKSGLEQNRDVAFYSMHPRYTASTWEDDIAL
TRY5H    GVVGI--MSLVEYRIVAGEHSLSVDSGLEQIRAVSSIVTHPNFDDITYEDDISL
TRY5J    CVDGA---NVARLRVVVGEHSQTQASGLEQISAVSSFTMHENYSSSTYENDISL
TRY5M    CVSGI--D-DTSLRVVAGEHSLSTDSGYEQDAFPVSFIVHEEYDTDHFKNDIAL
TTC      CTDGL--T-AAQLSIRAGTSSRGS---GGQVSSVARIYQHSSYNSRTIDYDISI
ChBT     CGV------TTSDVVVAGEFDQGSSSEKIQKLKIAKVFKNSKYNSLTINNDITL
ChPO     CNV------RTSHRVILGEHDRSSSAEDIQVMKVGKVFKHPRYNGYTINNDILL

cons                                                       *  :
```

```
PSS     LKLA-EPLDL---TPTAVGSICLPSQNNQ---EFSGHCIVTGWGSVR--EGG--
TAAe    LELE-ETVTF---S-DSCAPVKLPQKDTPVN--EGTCLQVSGWGNTQ--NPS--
TAFi    LKLL-NSIII---G-GNVQPVGLPFPNAEVD--EGVMATVSGWGTTS--AGG--
TAS     LELE-SELTF---S-NAVQPIALPEQDEPV--VDGTMATVSGWGNTQ--SAT--
TBT     IKLS-TPAVI---NA-RVSTLLLPSACA----SAGTECLISGWGNTL-SSGV--
TSS     IKLS-KPATL---NT-YVVQPVALPTSCA----PAGTMCTVSGWGNTM-S-ST--
TDM     IRLS-SSLSF---S-SSIKAISLATY----NPANGASAAVSGWGTQS--SGSS-
TLS1    LHLE-NPLEL---ND-KVAKIAMPDQDQEFEG----EAVISGWGTTF--SGA--
TLS7    LKLK-SSLHF---N-EKVKPIALPEKDQEF---IG-DVVVSGWGTIS--SSGP-
TNV     LQLE-SPLAL---G-SKIQPIELAEAADYYS--TGSKASVTGWGVEE--SSG--
TPC     LQFA-SPLTF---N-SFVGPIALPAQGQ----VASGDCTCTGWGTTT--EGG--
TPV     LQLS-QPLSF---N-DFVAPIALPEAGH----AASGDCIVSGWGTTS--EGG--
TPL     LKLA-TPLTF---N-NNVAPIALPAQGH---TATG-NVIVTGWGTTS--EGR--
TRY1    LKLK-GSIYN---VTESFIAVSLPSES---FQPSG-DCVVSGWGYQR-ANAG--
TRY2    IRLN-GAFVF---N-SFLKQVKLPRSGYFT--FPDTAVTVAGWGTTK--EGG--
TRY3    IKLI-TPLDF---SSGKVSPISLPAMGQE--TAAGTNVTVTGWGNLE-FNNP--
TRY4A   LKLS-SPLDY---STGKVGAINLPAQGATIE--TGTTCLVTGWGTTIAGGGG--
TRY4B   LELS-SPLTL---N-SVVGVVRIPAQGAQ--TAVGTNCVVSGWGTTS--SGGS-
TRY5A   VKLS-TPLVY---T-SKVAPVVLPPPLSELDPPAGLDVTIAGWGDTV-EVNG--
TRY5B   IYLA-SPLDL---SVPSAKAVNMPPPTSEFDPPAGTITTVSGWGTTS--SGG--
TRY5K   IYLA-SPLDL---SVPSAKAVNMPPPTSEFDPPAGTITTVSGWGRIS--TGG--
TRY5C   IYLA-APLDL---SVPSAKPINMPPATWEFDPPAGIVATVSGWGTTS--SGG--
TRY5D   IYLS-GPLNVLPFN-EAVQPINLAL-TGTADPEPGLSVTVAGWGTTS--ASG--
TRY5E   LFLE-SPLDL---SVPAARPILLPPPTEEFDPPSGLLLTVSGWGTTS--LGG--
TRY5F   LFLD-APLDL---SVPEAQPVLLPPPTSELDPPAGTIVTVSGWGTTS--SGG--
TRY5L   LFLD-APLDL---SVPEAKPVLLPPPTSELDPPAGTIVTVSGWGTTS--YGG--
TRY5G   IFLD-SPLDL---SVPSAKAIALPPSTSELDPPAGTVVTCSGWGTTS--SGG--
TRY5I   IVLT-EPFDL---SVPSAKPVNLPPPTSEFDAPAGTVFTVSGWGTTR--YGGI-
TRY5H   IFLT-EPLDL---SVPSAKPIALPGPTAEFDPPAGFRVNVSGWGTTT--SDG--
TRY5J   IFVA-TPFDL---SVASARPVNLPAPTSEFDPPAGTIITVSGWGTTS--EGG--
TRY5M   IFMNQAPLDL---SVPSAKPINLPEPTSQYDPPAGTHVNVSGWGFTN--SASWS
TTC     LELS-SSLTL---G-SNVSPVSLPSSSTSWN--AGTSVLVTGWGTTS--EGSS-
ChBT    LKLS-TAASF---SQ-TVSAVCLPSASDDF--AAGTTCVTTGWGLTR-YTNA--
ChPO    IKLA-APAQM---NM-RVSPVCVAETSDNF--AGGMKCVTSGWGLTR-HNAP--

cons    : .                 : :.                :***
```

```
PSS     --NSPNILQKVSVPLMTDEECSEY-Y---------NIVDTMLCAGY-AEGGKDA
TAAe    --ESSEVLRAAYVPAVSQKECHKA-YLSF-----GGVTDRMVCAGF-KEGGKDS
TAFi    --SLSDVLLAVNVPVISDAECRGA-YGET------DVADSMICAGDLANGGIDS
TAS     --ESNAVLRAANVPTVNQQECEKA-YGQT-----PGITDQMLCAGY-QQGGKDA
TBT     --NYPDLLQCLVAPLLSHADCEAS-YPG-------QITNNMICAGF-LEGGKDS
TSS     --ADSNKLQCLNIPILSYSDCNNS-YPG-------MITNAMFCAGY-LEGGKDS
TDM     --SIPSQLQYVNVNIVSQSQCASSTYGYG-----SQIRNTMICAA---ASGKDA
TLS1    --PPSFLLRWAKVNIVSKAECQNA-YGS-------RIDDSMICAAA---PGKDS
TLS7    ---ASPVLKAVTVQVVSDEDCSDA-YYG-------SIDETMICAAA---PGKDS
TNV     --ELSNYLREVSVPLISNSECSRL-YGQR------RITERMLCAGYVGRGGKDA
TPC     --YSSDALLKVTMPIVSDADCRAS-YGES------DIDDSMICAGV-PQGGKDA
TPV     --STPSVLQKVSVPIVSDDECRDA-YGQN------DIDDSMICAGM-PEGGKDS
TPL     --NTPDVLQKVTIPLVSDAECRSD-YGAD------EIFDSMICAGV-PEGGKDS
TRY1    --TTPDHLMAANVTILADEDCQNR-FSP-----HYKIYPGMICAGG-KE--KDA
TRY2    --NLSNVLLKTTVPVVSDEDCRLI-YGAG------LIVDSMLCAGY-TSGGYDS
TRY3    --NRPDVLNKVGIPTVSDLECRRS-YGPI------DMFDSFICAGV-PEGGLDS
TRY4A   --SVSDVLRKAAVPIVSNSDCNSM-YGGN------SILPSMLCAGF-VAGGIDS
TRY4B   --TIPDILRKVTVPIVSDDTCRGS-YGTN------AITDSMICAGF-RLGGADS
TRY5A   --TSSDVLISATVPIVADDVCNLA-YGGNEN--STIVFPDMMCTG---NGNADA
TRY5B   --SISNVLLSVDVPIVSDSDCNAA-YAGVFD--PNPIFPSMLCAGG-PPGGVDS
TRY5K   --SISNVLLSVDVPIVSDSDCNAA-YAGVFD--PNPIFPSMLCAGA-PAGGVDS
TRY5C   --SISNVLLSVDIPIISDDACNAI-YTDESEPKPKAIYESMLCAGG-PNGNVDS
TRY5D   --DRSDVLLSVDIPVVSDEDCNAA-YSGEYN--TDPVKPSMMCAGG-APGGLDA
TRY5E   --VISDQLRSVDVPVVDDATCNYA-YGGSTQ--NPDVFPSMICAGDMSAVQTFQ
TRY5F   --VISDQLRSVDVPVVDDDVCNRA-YGGTAA--SPQVYPSMLCAGDISNGGIDS
TRY5L   --AASDELRSVDVPVVDDDTCNRA-YGGTAA--NPEVFPSMMCAGDTSNGGIDS
TRY5G   --SISDVLRSVDIPVVSDADCDAA-YGGTAS--SPSVYPSMICAGDTTNGGIDS
TRY5I   --LVSDVLLSVDVPVVADVDCDAY-YGGTSQ--KPEVYPSMLCAGNTTDGGIDA
TRY5H   --DVSDVLRSLEVPVIPDFDCDAA-YGGDGV--YIAVFPSMVCAGDTVTSRIPY
TRY5J   --SVSDTLLSVDIPVISDADCNTA-YGGN------AVFSSMMCAGGP-NGGIDS
TRY5M   DDRLSDLLRSVNISVVSDQVCDKA-YGGDSE--NRIVLPSMMCAGDMKKGGLDS
TTC     --SLPSALQGVTVQIVSQSTCSSA-YGSGS------ITGRMLCAG-VTNGGKDA
ChBT    --NTPDRLQQASLPLLSNTNCKKY-WGT--------KIKDAMICAGA---SGVSS
ChPO    --DTPALLQQAALPLLTNDDCRRY-WGN--------KISNLMICAGA---SGASS

cons           *       :    *    :          :   :.*:.
```

```
PSS      CQGDSGGPLVC----PNGDGTYSLAGIVSWG--IGCAQPRNPGVYTQVSKFLDW
TAAe     CQGDSGGPLVH--------DN-TLVGVVSWG--YGCAQAGYPGVYARVASVRDW
TAFi     CQGDSGGPLYM----GS-----TIIGIVSWG--YGCAYAGYPGVYTQVSYYVSF
TAS      CQGDSGGPLVA--------DG-KLIGVVSWG--FGCAQAGYPGVYSRVASVREW
TBT      CQGDSGGPVAC--------NG-QLQGIVSWG--YGCAQKGKPGVYTKVCNYVDW
TSS      CQGDSGGPVVC--------NG-ELQGVVSWG--YGCAEPGNPGVYAKVCIFNDW
TDM      CQGDSGGPLVS----G----G-VLVGVVSWG--YGCAYSNYPGVYADVAVLRSW
TLS1     CQGDSGGPMVC--------DG-VQCGIVSWG--YGCADPKYPGVYAKLSKFMDW
TLS7     CQGDSGGPLAQ--------DG-TLVGIVSWG--YGCAAPGYPGVYGKVSKFIDW
TNV      CQGDSGGPLVQ--------DG-KLIGIVSWG--FGCAEPNYPGVYTRVTALRSW
TPC      CQGDSGGPLAC----SDTGSP-YLAGIVSWG--YGCARPNYPGVYCEVAYYVDW
TPV      CQGDSGGPLAC----SDTGST-YLVGIVSWG--YGCARPNYPGVYAEVSYHVDW
TPL      CQGDSGGPLAA----SDTGST-YLAGIVSWG--YGCARAGYPGVYTEVSYHVDW
TRY1     CQGDSGGPLVCKSS---TGQN-VLTGIVSWG--IGCATPTIPGVYTKVAKYMDW
TRY2     CQGDSGGQLML----G---DK-TLVGIVSWG--KGCGQPDYPGVYTEVSAYIGW
TRY3     CNGDSGGPLFT----SDPQ---QLIGIVSWG--KDCALPGYYGVYTEVSYFSDW
TRY4A    CQGDSGGPLVT----LN--PN-VLVGVVSWG--NGCANPGYPGVYTRVASFTNW
TRY4B    CQGDSGGPLV------DEGTN-LLIGVVSWG--IGCADAGYYGVYTEVSYFHNW
TRY5A    CNRDLGGPLFT----GTGAEA-VIHGIISWG--IGCGASSYPDAYTQVSYFLDW
TRY5B    CQGDSGGPLFT----GTGADA-VQHGIVSWG--IGCAQAAYPGVYTQVSYYLDW
TRY5K    CQGDSGGPLFT----GTGADA-VQHGIVSFGPSAGCALAEYPGVYTQVSYFLDW
TRY5C    CQGDSGGPFFI----GTGTNA-IQLGVVSWG--RGCATGHYPGVYTQISYFLDW
TRY5D    CQGDSGGPLFVLPADGNVANV-QQLGIVSWG--RGCGLADYPGVYTQLSYYLEW
TRY5E    VELISGGPLFLLPLDGNVTEA-RQIGVVSWG--RGCAISAFPGVYTQVSYFLDW
TRY5F    CQGDSGGPLFT----GTGASA-VQHGIVSWG--QGCALAGYPGVYTQVSFFLDW
TRY5L    CQGDSGGPLFT----GTGASA-VQHGIVSFG--QGCALAAYPGVFTQVSFFLDW
TRY5G    CQGDSGGPLFT----GTGETA-VQHGIVSWG--QGCALARFPGVYTQVSYFLDW
TRY5I    CQGDSGGPLFS----GSGETA-VQHGIVSWG--KSCAEPQWPGVYTQVSYFVEW
TRY5H    CQGDSGGPLFT----GTGETA-VQHGIVSWG--QSCTYSQFPGVYTQVSYYLDW
TRY5J    CQGDSGGPLFT----GTGETA-VQHGIVSWG--QGCAQAAYPGVYTQVSYFLDW
TRY5M    CQGDSGGPLFT----GSGETA-VLYGIVSWG--EGCALASYPGIYTQVSYFLDW
TTC      CQGDSGGPLVV----GN-----VLAGIVSWG--AGCGRPSYPGVYSNVPALRSF
ChBT     CMGDSGGPLVC----KKNGAW-TLVGIVSWG--SSTCSTSTPGVYARVTALVNW
ChPO     CMGDSGGPLVC----QKAGAW-TLVGIVSWG--SGTCTPTMPGVYARVTELRAW

cons          ** .                ::*:*    .       . :  :     :
```

```
PSS       IRNTNIDGSNVIEFI-----------------------I
TAAe      VKEVSG--------------------------------L
TAFi      IKSV-----------------------------------
TAS       VRENSG--------------------------------V
TBT       IQETIAAN------------------------------S
TSS       LTSTMASY-------------------------------
TDM       VVSTAN-------------------------------SI
TLS1      VKENV----------------------------------
TLS7      IVEHK----------------------------------
TNV       ISEIAG--------------------------------L
TPC       VLANSS---------------------------------
TPV       IKANA----------------------------------
TPL       IKANAV---------------------------------
TRY1      INKIMA-------------------------------DN
TRY2      INMKLSTAEA--------------------SNNRNI--S
TRY3      IAATIAAADA----------------------------H
TRY4A     IKTNAV---------------------------------
TRY4B     IVSFAG---------------------------------
TRY5A     IAANRG---------------------------------
TRY5B     IMANRG---------------------------------
TRY5K     IAANKQ---------------------------------
TRY5C     INENLH---------------------------------
TRY5D     INQNMV-------------------------------PA
TRY5E     IAANRV---------------------------------
TRY5F     IAANRV---------------------------------
TRY5L     IATNRV---------------------------------
TRY5G     IAAARG---------------------------------
TRY5I     IALAMQ---------------------------------
TRY5H     IAANRR---------------------------------
TRY5J     IAANKI---------------------------------
TRY5M     IAANQRVYSNYFNWPHLRHLSTRPMPTPDSKHVRTVSTS
TTC       IQQTTG--------------------------------I
ChBT      VQQTLAA-------------------------------N
ChPO      MDQTIAN-------------------------------N

cons      :
```
