# Supplementary material for: Acclimatory responses of the Daphnia pulex proteome to environmental changes. II. Chronic exposure to different temperatures (10 and 20°C) mainly affects protein metabolism
Source: BMC Physiol. 2009 Apr 21;9:8. doi: 10.1186/1472-6793-9-8 (PMC2678069; doi:10.1186/1472-6793-9-8)
Supplement: Additional File 2 — Multiple sequence alignment of chymotrypsin-like sequences. The multiple-sequence alignment was performed using the T-Coffee algorithm [54]. NCBI accession numbers for the symbolic sequence names are listed in the Figure legend 6. [file 1472-6793-9-8-S2.pdf]

T-COFFEE, Version_5.49Wed Dec 19 12:12:01 2007

Cedric Notredame

CPU TIME:16 sec.

SCORE=55

*

BAD AVG GOOD

*

CES	:	59
CPC	:	60
CUP	:	60
ChPV	:	60
CHL	:	54
CHY1A	:	61
CHY1C	:	61
CHY1B	:	56
CHY1D	:	61
CHY1E	:	59
ChAG	:	48
ChAM	:	44
ChBT	:	59
ChPO	:	57
ChCF	:	41
ChPC	:	49
ChPH	:	50
ChRP	:	46
JoDM	:	48
TBT	:	57
TSS	:	57
CHY1F	:	58
CHY1G	:	56
CHY1H	:	59
cons	:	55

```
CES      ----------------------L-LLALVAAA-SA-----A-------EWRWQ
CPC      --------------M---IATLSL-LLACVAVA-SGNPAVGK-------PWHWK
CUP      --------------M---IVKLAL-ILVCVALA-SGNPAAGT-------EWRWK
ChPV     --------------------------LVCVAVAASGNPAAGK-------PWHWK
CHL      -------------------MK----FLLVFALALATTSAFQH-------PASIF
CHY1A    ------------------MKVLAI-VLVAFVVA----QVAAR-------DLSKY
CHY1C    ------------------MKVLAI-VLVAFVVA----QAAAW-------DLSKY
CHY1B    --------------MN--VSAAVV-LLVAIVFA----QAAAR-------DLARY
CHY1D    ------------------MKVFAI-VFVAIAVA----QASGL-------DLSKY
CHY1E    ------------------MKFLAAALLFAVVVA----QVAAR-------DLSKY
ChAG     MRIKDAAGATSQYTTTVRMKTFAL-LVALFAVA----SAEWI-------DIDWS
ChAM     ------------------MKL-SL-IILAVALC----EARPR-------D--VD
ChBT     ------------------------------------------------------
ChPO     ------------------MGI-----LSCLAFA----GAAYG------------
ChCF     ------------------MKII-V-ALCLVAFV----ACESV-------PTF--
ChPC     ------------------MKVALV-VLALFGVS----LAASI-------D--NI
ChPH     ------------------MKGFFA---LCFIIA----TAVLA-------EAYYP
ChRP     ------------------MKTVAV-FLCVIAIV----LAE--------------
JoDM     ------------------MKLF-V-FLACLAVA----SAGVV-------PSESA
TBT      ------------------MHPL-L-ILAFVGAA----VAFP-------------
TSS      ------------------MISL-V-FVLLIGAA----FA---------------
CHY1F    ------------------MKFLAL-VLALAVAA----QAAVNM------PHKKI
CHY1G    ------------------MKFLVA-VLALAVLS----QAAEIP------MRRNL
CHY1H    ------------------MKIAAL-ILVAVALA----QAAEIPKLRNFRNPANL

cons

CES      FRHPTVTPNPRA-KNPFRVTKSSPV-QPPAVRGTKAVENCGPV--A-PRNKIVG
CPC      SPKPLVTP-------------------------------IGPV----KSQRIVG
CUP      SPKPLMTP-------------------------------IGPV----KSSRIVG
ChPV     SPKPLVDA-------------------------------RGPAP-N-AGAKIVG
CHL      EL---------------------------------------------REGRIIN
CHY1A    QPRSVLYPRLPS-KNTNSF---VPI-KRSATVNTRGFC--GQAN-V-TSSRIVG
CHY1C    QPRSVLFPRAPT-KNTNSF---VPV-KRSPTVDTRGFC--GQAK---SASRIVG
CHY1B    KPRGVLFPRPPS-RNKKPI---FPV-KHTATINTRGFC--GREN-S-TNSRIVG
CHY1D    QPRSVLYPRAPV-ENPNRW---VPP-KVAPTVDTRGFC--GQVQ-S-APSRIVG
CHY1E    QPRSVLFPRPPV-TQKNQY---VPV-KRAATVDTRGFC--GTAK-TSNQDRIVG
ChAG     QVRPI--------EEFDHYWAR----LPAELQ-------VYRTK-L-PSHRITN
ChAM     -------------------------------L-------TARAS-V-AAPFIIN
ChBT     ---------------------------CGVPA-------IQPVL-S-GLSRIVN
ChPO     ---------------------------CGSPA-------IPPVI-T-GYSRIVN
ChCF     -VHPN--------AE------------LIAQA-------KNMMT-Q-KSSRIVY
ChPC     EIPPS--------KN-----------IYVEPI-------NQPEV-D-PSLEIVN
ChPH     EAPLR--------AG-----------L------------------R-RRPKVVG
ChRP     -------------------------------------------G-V-LDKRIVN
JoDM     RAVPV--------KD-----------M-------------PRAG-K-IEGRITN
TBT      -------------------------------------------S-D-DDDKIVG
TSS      ---------------------------------------------T-EDDKIVG
CHY1F    FPRGELFPRAQVPQGPGYF---TPT-KVAPTVDTRGFC--GQSK-V-DSSRIVG
CHY1G    RPRSELFPRAPVAPGPGHI---VVT-EPAKPVDMRGFC--GQAK-V-DSSRIVG
CHY1H    PPRSALFPRPSQVQGPGYF---VPTGEHAHVFSTRGVC--GQVKNQ-ESERIVG

cons                                                        :
```

```
CES      GMEVTPHAYPWQVGLFID----D---MYFCGGSIISDEWVLTAAHCMDG-----
CPC      GQEATPHTWVHQVALFID----D---MYFCGGSLISSEYVLTAAHCMDG-----
CUP      GVEAVPNSWPHQAALFID----D---MYFCGGSLISPEWILTAAHCMDG-----
ChPV     GTEAVPHSWPHQVALFID----G---MYFCGGSLISSEWVLTAAHCMDG-----
CHL      GYEAYTGLFPYQAGLDITLQDQR---RVWCGGSLIDNKWILTAAHCVHD-----
CHY1A    GTEAVPNSLPWQVALFID----D---QYFCGGSLISNEWVLTAAHCADA-----
CHY1C    GTEAVPNSLPWQVALFID----D---KYFCGGSLISNEWVMTAAHCADG-----
CHY1B    GTEAEPHSLPWQVALFVD----D---RFFCGGSLISNEWILTAAHCAED-----
CHY1D    GTEATPHSAPWQVAIFID----G---QYFCGGTLISNEWVLTAAHCADN-----
CHY1E    GTEAVPNSRPWQVALFID----D---QYFCGGSLISPDWVLTAAHCADN-----
ChAG     GQEATPGQFPYQIALLSNFA-TG---TGLCGGSVLTNNYILTAAHCVISGATTL
ChAM     GSPADISNFPYQCSLRY----AG---SHTCGCSVLNAGVVLTAAHCVDGRV---
ChBT     GEEAVPGSWPWQVSLQD-K--TG---FHFCGGSLINENWVVTAAHCGVT-----
ChPO     GEEALPHSWPWQVSLQD-Y--TG---FHFCGGSLINENWVVTAAHCNVR-----
ChCF     GDDAQISDFKWQASLRR----SG---SHICGAAIVSDKHAITAAHCVDGTS---
ChPC     GQEVVPHSIPYQIFLVA-SAGET---SWTCGGSLITKRYVLTAAHCIQG-----
ChPH     GTEAVPHSVPYQLGLLL-N-------GSFCGGSLITKRFVLTAAHCGVV-----
ChRP     GEKAELYAHPHQASLQL-FQ-DSHGWYHICGAVLVGPNKLVTAAHCVQGQD---
JoDM     GYPAYEGKIPYIVGLS--FN-DG---GYWCGGSIIDHTWVLTAAHCTNS-----
TBT      GYTCAENSVPYQVSL----N-AG---YHFCGGSLINDQWVVSAAHCYQY-----
TSS      GYECKAYSQTHQVSL----N-SG---YHFCGGSLVNENWVVSAAHCYKS-----
CHY1F    GEEAIPHEFPWQVSVLID----G---SGFCGGSLISPDWVLTAAHCADG-----
CHY1G    GVEAVPHEFPWQVAVTID----G---SSFCGGTLISDEWVMTAAHCADG-----
CHY1H    GVEAVPHEFPWQVALVVD----N---SWFCGATIISADWILTAAHCTDG-----

cons     *             :              **  ::     ::****

CES      AGFVEVVMGAHSIH--DETEATQ--VRATSTDFFTHENWN--SFTLSNDLALIK
CPC      AGFVEVVMGAHNIR---EDEASQ--VSMTSTNFAIHENYN--AFTITNDVAYIR
CUP      AGFVDVVLGAHNIR---EDEATQ--VTIQSTDFTVHENYN--SFVISNDIAVIR
ChPV     AGFVDVVMGAHNIR---ENEDTQ--VTMTSTDFFTHENWN--SFLLTNDLALIR
CHL      AVSVVVYLGSAVQY------EGE--AVVNSERIISHSMFN--PDTYLNDVALIK
CHY1A    AVFFDIYLGSHNVRLTAAEEPTR--VEIRSTQYTVHPDWA--SLRIRNDVALIK
CHY1C    AVFFNILLGSHNVRLAAADEPTR--VEVTSFDYTVHPDWA--SLRIRNDVALIK
CHY1B    AGFFDILLGSHNVRLNATEEPSR--FEIRSNVSIVHPGWS--SFRFMNDIALVK
CHY1D    AIFFNIYLGSHNVRLTAAEEPTR--VEVRSTQYTVHPNWG--SIRIINDVALIK
CHY1E    ATFFNIMLGAHNVRLTAAEEPNR--VEYRSEVYTIHPNWG--STLLRNDLALIK
ChAG     ATSGTAIMGAHNRNV---NEPTQQRIGFTSAGIRAHPGYN--PTNIRNDIAVVR
ChAM     ATAFSVLAGSTDRTV-----SGD--IDASGFTMNSAYDGN--AGGFPNDIATVA
ChBT     -TSDVVVAGEFDQGS---SSEKI--QKLKIAKVFKNSKYN--SLTINNDITLLK
ChPO     -TSHRVILGEHDRSS---SAEDI--QVMKVGKVFKHPRYN--GYTINNDILLIK
ChCF     ASSLSLRVGSSYH----KNGGTI--VGVQT--IRVHERYNGNAPGYPNDIAILV
ChPC     AKSVHVTLGAHNLAK---HEASK--VTVNGRSWVIHEKYD--STNIDNDIGVIQ
ChPH     TKHPVVVMGAHKITE---KEPNQ--VAMTGKNVVVHKQYS--PNTLRNDIALVE
ChRP     ATKLRVEVGALNLLD--PPNAYE--QTIPVEFFIIHPLYNEKGNAYPNDIAILY
JoDM     ANHVLIYFGAS-FRH---EAQYT--HWVSRSDMIQHPDWN--DF-LNNDIALIR
TBT      --HIQVRLGEYNIDV---LEGGE--QFIDASKIIRHPKYS--SWTLDNDILLIK
TSS      --RVEVRLGEHNIKV---TEGSE--QFISSSRVIRHPNYS--SYNIDNDIMLIK
CHY1F    AGRFLITLGDHDRT---VTEPSQ--IAVTTTTYTVHPGWN--SATLADDLALIR
CHY1G    ANRFTLLLGAHDRT---VAEPSQ--LTIQTTVHATHPNWN--PSTLANDVALIR
CHY1H    GRSFEVILGAHNKN---IVESTQ--VRITATQFTMHPKWN--SAKLQNDVALIK

cons             *                                      :*:  :
```

```
CES    MPAPIEF-NDVIQPVCL-PTYTDASDDFVGESVTLTGWGKPSDSAF-GIAEQLR
CPC    LPSPVTF-DANIGAVAL-PT-SDPA---VGTTVTPSGWGMDSDSAF-GISDILR
CUP    LPVPVTL-TAAIATVGL-PS-TDVG---VGTVVTPTGWGLPSDSAL-GISDVLR
ChPV   LPNAVEF-TDNISTVKL-PS-SDVA---VGTTVTPTGWGRPSDSTG-SISNVLR
CHL    IP-HVEY-TDNIQPIRL-PSGEELNNKFENIWATVSGWGQSN-----TDTVILQ
CHY1A  LPAPIEF-TPEIQPVCLAPT-SETD--HVDDILHISGWGKPSDAAA-GISPVLR
CHY1C  LPAPIEF-TPEIQPICMAPS-TEPD--HVGDILHNSGWGKPSDAAA-GISPTLN
CHY1B  IPQPIKF-TPEIQPVCLAPP-SEPD--HVDDILHVSGWGRHSELTP-GISQVLR
CHY1D  LPAPIEF-TPEIQPICMAPS-TEPD--HTGDILHISGWGKPSDAAA-GISPVLR
CHY1E  LPESVTF-TDAISPICLAPA-TEPL--HAGDPLHVSGWGKPSDAAT-GISPVLR
ChAG   LNSPITF-TARIQPIRLPG-RSD-SRQFGGFTGTVSGFGRTTNTG--ATSPVVM
ChAM   LTSNLNLGDPNIAAASLPP-NNN--DQFVGSQCTITGWGRTGTS-N-ILPATLQ
ChBT   LSTAASF-SQTVSAVCLPS-ASD--DFAAGTTCVTTGWGLTRYTNA-NTPDRLQ
ChPO   LAAPAQM-NMRVSPVCVAE-TSD--NFAGGMKCVTSGWGLTRHNAP-DTPALLQ
ChCF   VAGSLTS-NVNAEAVDLPQ-NPN--ENYNGADCEITGWGRTE--LG-GLPDILQ
ChPC   LERNLTL-TRSIQLARLPS-LRDVGINLEGRTATVSGWGLTNGIFQ-TTTDVLR
ChPH   LPEDAPL-SQYVQLVKLAA-VDA--GLFVGETARVSGWGRAYDSST-TISPVLR
ChRP   LSSPVTY-NKNVQPAELAP-KG---SSFANEQCIITGWGRTIGG-G-PTAAHLK
JoDM   IPH-VDF-WSLVNKVELPS-YNDRYNSYSGWWAVASGWGLTDN-NS-GMSNYLN
TBT    LSTPAVI-NARVSTLLLPS-ACA----SAGTECLISGWGNTLSSGV-NYPDLLQ
TSS    LSKPATL-NTYVQPVALPT-SCA----PAGTMCTVSGWGNTMS-ST-ADSNKLQ
CHY1F  LPSPVTL-TPEIQPICLAPA-TEPT--HVGDTLLVSGWGKTADGVLQGISDVLM
CHY1G  LPEPITF-SPEISPLCMSPA-TEPD--HVGDTLLVSGWGKTADGAFQSISPVLM
CHY1H  LPRPVSF-TPEIAPICLAPN-TESD--HVGDILLASGWGLDSDSAS-STVANLR

cons   :               :            .     :*:*             :


CES    EVDVTTITTADCQAY----YG--IVTDKILCI----D-SEGGH-GSCNGDSGGP
CPC    QVDVPIMSVVDCDAV----YG--IVSNNQICI----D-STGGR-GTCNGDSGGP
CUP    QVDVPIMSNADCDAV----YG--IVTDGNICI----D-STGGK-GTCNGDSGGP
ChPV   QVDVPIMTNDDCNAV----YG--IVGNGVVCI----D-SEGGK-GTCNGDSGGP
CHL    YTYNLVIDNDRCAQE---YPPG-IIVESTICG----D-TCDGK-SPCFGDSGGP
CHY1A  EVDAPCISNEECALT----YGN-TIQPGNICV----N-TTGGH-SSCNGDSGGP
CHY1C  EVYMPCMSNDECAAY----FGN-TIQPGNICT----D-TTGGH-SACNGDSGGP
CHY1B  EIDVPCISNEECALT----YGE-TITDGIICI----S-TAGGR-GSCNGDSGGP
CHY1D  EVDVPCISNAECAAT----YGA-TITAGNICV----D-TTGGK-GSCNGDSGGP
CHY1E  EVDVVGITNQECVAA----YSILTIRDSNLCV----S-TLGGK-SSCNGDSGGP
ChAG   FTSNPVMTNADCIA----RWNTALIQPQNVCL----S-GDGGR-SSCNGDSGGP
ChAM   QVTMPIISNAECASRMSSVSG-ANVNDGHICI------YNGDS-GSCNGDSGGP
ChBT   QASLPLLSNTNCKK----YWG-TKIKDAMICA----G-ASGV--SSCMGDSGGP
ChPO   QAALPLLTNDDCRR----YWG-NKISNLMICA----G-ASGA--SSCMGDSGGP
ChCF   VANTNVLSQSECTN----YWG-SNINTGHVCV------RTGNN-GACQGDSGGP
ChPC   -ANNTIISNKECND----VF--KIVQPTEVCL----S-IAGGR-SACSGDSGGP
ChPH   VVESNILTNEECRK----RFG-FAVFKSVICL----D-GSQKK-SSCNGDSGGP
ChRP   QAYISKITRSQCNLRW-ALYG-QLITDKHICVYEASD-PAGTRPSACQGDSGGP
JoDM   CVDVQIIDNNDCRN----YYGSNYITDNTICI----N-TDGGK-SSCSGDSGGP
TBT    CLVAPLLSHADCEA----SYP-GQITNNMICA----GFLEGGK-DSCQGDSGGP
TSS    CLNIPILSYSDCNN----SYP-GMITNAMFCA----GYLEGGK-DSCQGDSGGP
CHY1F  KVTAPGITTADCAAT----YGD-IITDNILCI----D-TTGGK-GSCNGDSGGP
CHY1G  KVTAPAISTADCAAT----YGD-IITDNILCI----D-TTGGH-GSCNGDSGGP
CHY1H  KVNAPGISSADCRAV----YGN-NVQDTVLCI----D-TTGGH-GTCNGDSGGP

cons         :    *            :    .*             ..* ******
```

```
CES      MNYVTGG-VTQTRGITSFGSST---GCETGYPDGYTRVTSYLDWIESNTGIA-I
CPC      LNY--GG-T--TVGITSFGASA---GCEAGYPDAFTRVSAYLDWIQANTGVT-P
CUP      LNY--NG-L--TYGITSFGAAA---GCEAGYPDAFTRVTYFLDWIQTQTGIT-P
ChPV     LNL--NG-M--TYGITSFGSSA---GCEAGYPDAFTRVYYYLDWIQQKTGVT-P
CHL      FVLSDKN-L--LIGVVSFVSGA---GCESGKPVGFSRVTSYMDWIQQNTGII-F
CHY1A    LTFVNGG-VHNQVGIVSFGSSA---GCEVGYPAAFARVSYFAEWISSVTGLL-I
CHY1C    LSYINGG-VYNQVGIVSFGSSA---GCEVGYPAAYTRVSYYADWISSVTGLV-I
CHY1B    LTFVNDG-VHNQVGVVSFGSSM---GCDKNLPAGFSRVSFYAEWISSITGLI-I
CHY1D    LTYVANG-VHNQVGVVSFGSSA---GCEVGLPAGFSRVSYFAEWISSVTGLV-I
CHY1E    LSFINNG-VYNQVGVVSFGSNQ---GCELEIPAGFARLSSFSDWISSVTGLV-I
ChAG     LTVQDG--GSLQIGIVSFGSAA---GCSIGMPSVYARVSFYLDWIDANSDFN-A
ChAM     MNC--QG---YVAGVTSWGISSALGNCMVSYPSVYTRTSYFLSWIANNS-----
ChBT     LVCKKN-GAWTLVGIVSWGSS----TCSTSTPGVYARVTALVNWVQQTLAAN--
ChPO     LVCQKA-GAWTLVGIVSWGSG----TCTPTMPGVYARVTELRAWMDQTIANN--
ChCF     LTC--SG---VLVGVTSWGYS----DCRVSHPSVYTRITTFLDWINDNMSR---
ChPC     LVIDN-----VQHGIVSYGSSY----CR-STPSVFTRVSSYLNWLQTHSEWRAQ
ChPH     LVVKTE-EGEVQVGVVSYGSSA---GCEKGFPAGFSRVTSFVDWVKDNSDYT-D
ChRP     LMC--GADFKLLAGVTSWGLA----SCTGGMPSVYTRVSEYVDWVEAN------
JoDM     LVLHDNN---RIVGIVSFGSGE---GCTAGRPAGFTRVTGYLDWIRDHTGIV-Y
TBT      VAC--NG---QLQGIVSWGYGC----AQKGKPGVYTKVCNYVDWIQETIAAN-S
TSS      VVC--NG---ELQGVVSWGYGC----AEPGNPGVYAKVCIFNDWLTSTMASY--
CHY1F    LSFVNAG-VYNQVGIVSFGARA---GCAAGFPAGFTRISSYTQWISDTTGLI-F
CHY1G    LSFDNNG-VYNQVGIVSFGSSA---GCTRGLPAGFTRVSSYAQWISLVTGLV-I
CHY1H    LSYINNG-VYNQVGLVSFGSAS---GCELGYPTGFSRISSFVDWIVSVTGLV-V

cons     .            *:.*:        .    *  :::      *:         


CES      DP
CPC      --
CUP      --
ChPV     --
CHL      --
CHY1A    --
CHY1C    --
CHY1B    --
CHY1D    --
CHY1E    --
ChAG     QP
ChAM     --
ChBT     --
ChPO     --
ChCF     --
ChPC     --
ChPH     --
ChRP     --
JoDM     --
TBT      --
TSS      --
CHY1F    --
CHY1G    --
CHY1H    --

cons
```
